# Impact of a Fulbright award: A bibliometric analysis of persistence

**DOI:** 10.4102/hsag.v30i0.2776

**Published:** 2025-03-05

**Authors:** Marie Hastings-Tolsma, Charlene Downing, Annie Temane, L. Amy Giles, Jean L. Hillyer, Sean C. Beatty

**Affiliations:** 1 Louise Herrington School of Nursing, Baylor University, Dallas, Texas, United States; 2 Department of Nursing, University of Johannesburg, Johannesburg, South Africa; 3 Department of Pathology and Laboratory Medicine, Faculty of Bioinformatics Scientist, University of British Columbia, Vancouver, British Columbia, Canada

**Keywords:** bibliometrics, bibliometric analysis, bibliometric mapping, altmetrics, infographics, data visualisation, citation analysis, Fulbright award

## Abstract

**Background:**

While hundreds of Fulbright awards have been given, little is known about the impact of such engagement despite a goal of increased research partnership and collaboration.

**Aim:**

The extent and impact of a Fulbright award was explored by examining referencing of primary collaborative publications.

**Setting:**

Seven databases and two alternative sources from 2013 to 2023 were reviewed.

**Methods:**

Co-citation analysis identified pairs of referenced articles. Subsequently, a bibliometric approach was used to quantitatively and visually capture and analyse publications using data visualisation software.

**Results:**

A search of sources found 773 citations citing the 16 primary works. Following the elimination of duplicates, 273 publications remained. Also examined was the non-scientific downstream noted in social media (*n* = 66). Based on co-citation analysis, there was a sharp uptick in the utilisation of primary citations (*n* = 273) compared to a 2019 analysis (*n* = 42). Journal Impact Factors of citing works had a high of 5.379 from 2.079 in 2019. Primary citations in open access journals demonstrated greater referencing, and the COVID-19 pandemic exacerbated utilisation of some works. Citing works focussed on three clusters: compassion fatigue, birth stories and Ubuntu. Most citing works originated from South Africa and the United States of America and consisted of multidisciplinary investigators with interfacility alliance and team science engagement. Nursing or midwifery were the main disciplines of first authors in citing articles (*n* = 153).

**Conclusion:**

Co-citation analysis and downstream use of publications in social media provided evidence of the impact of a Fulbright award on scholarship with persistence over time.

**Contributions:**

Fulbright awards promote collaborative teamwork between disciplines and is of clear benefit to scientists.

## Introduction

Fulbright awards, an international educational exchange programme, were initiated in 1946 through the efforts of Senator William Fulbright of the United States of America (USA) (U.S. Department of State Bureau of Educational and Cultural Affairs (ECA) 2023). Engagement offers the opportunity to increase awareness of cultural diversity through intensive immersion in teaching, research and/or community engagement while promoting partnership and collaboration.

Designed to increase understanding between those from the USA and other countries, hundreds of Fulbrights have been awarded though relatively little is known about the impact and reach of these engagements. Over the past couple of decades, there have been numerous reports regarding the experience of the Fulbright awardee and results from approved project work. However, there is a paucity of knowledge regarding the extent and the impact of the award over time, as well as whether collaborations are sustained following the exchange period. The need for such determination has been noted (Downing et al. 2021; Van Woerkom 2010).

One of the investigators (M.H.-T.) was a Fulbright USA Faculty Scholar to the Republic of South Africa (RSA). The research-teaching award was for 1 year (2012–2013) with the University of Johannesburg Department of Nursing hosting the scholar. The purpose of this project was to ascertain the extent and impact of the Fulbright award by examining primary publications that emanated from ongoing collaboration using bibliometric analyses. A secondary purpose was to examine non-scientific downstream citations as found in social media sources. Finally, investigators sought to compare dissemination efforts from works published between 2013 and 2019 with the 2013 and 2023 analyses. In summary, this work advances more traditional bibliometric analytic work specific to a focussed area of scholarship.

### Bibliometrics

Bibliometrics uses citations from publications to gauge the influence of these published works on other scientific publications (Bornmann & Haunschild 2018). The process uses statistical analyses to then provide a visual graphic (McBurney & Novak 2002). The approach has been deemed a reliable method to conduct quantitative and empirical investigation of previously published works in any field (Ellegaard & Wallin 2015), with research questions answered by extracting publication data (Belter 2015). The result is a type of infographic that gives a visual snapshot of complex data (Thompson & Walker 2015), enables scientists to conduct macroscopic and microscopic analyses of large numbers of publications (Alfonzo, Sakraida & Hastings-Tolsma 2014) and facilitates ease in understanding the impact of the data (Smiciklas 2012).

There has been an explosion of bibliometric works over the past 25 years, in part because of access to relatively easy-to-use mapping tools and support from academic librarians (Cox et al. 2019). For example, few studies have examined the use of bibliometrics in nursing or related health sciences with detailed content analysis until more recently. Kokol and Vošner (2019) reviewed the use of bibliometrics in nursing and demonstrated a dramatic recent increase with a positive trend in the number of publications, diversity of journals and number of countries (with greater production in G7 countries and those with successful economies). Internationally authored bibliometric publications were cited more than those written by authors from just one country. Findings were consistent with prior research (Kokol et al. 2014; Železnik, Blažun Vošner & Kokol 2017).

Scientists in the academic environment have been particularly interested in the use of bibliometrics as a means of documenting the impact and reach of scholarly works – beyond the more traditional metrics of Journal Impact Factor (JIF), Eigenfactor, m-quotient and h-, i10- or g-indices. Interestingly, universities (Ndwandwe et al. 2021) and grant funding agencies (Gunashekar, Wooding & Guthrie 2017; Recio-Saucedo et al. 2022) have increasingly used bibliometrics to assess scholarly output by faculty.

A corollary in bibliometrics that has received far less attention is how citations impact social media sources and lay publications – the ‘altmetrics’ (Garcia-Villar 2021). Web-based altmetric tools provide a more nuanced story about the use and impact of published works (Champieux 2015). Such data give evidence of how and where published works are shared and discussed and by whom, though meaning has not yet been fully clarified (Díaz-Faes, Bowman & Costas 2019). Altmetrics have gained increased acceptance across institutions and are increasingly used as a tool to document scholarship, supporting professional advancement efforts such as promotion and tenure. A robust evaluation of such works provides an important complement to traditional methods in determining scholarly output and impact (Baheti & Bhargava 2017) and gives evidence of heightened outreach and engagement (Wang et al. 2017). Determining varied social media metrics, such as downloads, reads, tweets, Facebook posts and news mentions, is increasingly doable as available tools (e.g. Altmetrics, Google Scholar, Plum X, ResearchGate and Mendeley) have proliferated. Caution needs to be exercised; however, as these alternative metric tools need relevance to research objectives (Bornmann & Haunschild 2018).

This research aimed to compile and analyse the scholarly articles that emanated from a Fulbright award. The research objectives were:

1. What were the number of published works citing the primary publications published between 2013 and 2023?
2. What was the growth trajectory in the number of publications citing primary works?
3. What were the journal distribution, JIFs, keywords, major subject clusters, geographic distribution of the primary author, disciplines and research methodology of studies citing primary, seminal works?
4. What were the non-scientific uses of primary citations as noted in social media?
5. What were the bibliometric mapping and non-scientific use of primary citation differences between the 2019 and 2023 analyses?

## Research methods and design

### Study design

A bibliometric analysis was conducted to examine the citation of articles emanating from the collaboration of researchers engaged in work as the result of a Fulbright award (*N* = 16) (Table 1). Citation analyses examined primary referring documents for citations and provided a means of assessing both publication output and the influence on the defined field. Specifically, co-citation analysis was used to track pairs of studies that were cited together in referent, source articles. When a pair of studies are co-cited by varied authors, clusters of research form. Co-citation analysis is prospective and dependent on the development of an academic field (Small 1973). Where citations were noted, it was believed to reflect confidence regarding the impact of identified publications (e.g. articles, books and other works) on science, as a whole (Belter 2015). Specifically, this research analysed the merit and impact where primary citations were sourced – a widely used bibliometric mapping approach (Schaer 2013). Further, PlumX and Altmetric were used to examine the non-scientific downstream use of primary publications in social media and lay literature.

**TABLE 1:** Primary referenced works (*N* = 16) with number and database location of secondary citations (*n* = 273).

Citation number	Primary referenced works	JIF	Open access	# Citing sources	Secondary citations by database*						
					CINAHL	Web of science	Scopus	Google scholar	PubMed	EMBASE	Researchgate
1	Austin, B., Downing, C. & Hastings-Tolsma, M., 2019, ‘Experience of neonatal intensive care unit nurses in providing developmentally-supportive care: A qualitative study’, *Nursing Health Science* 21(3), 336–344. https://doi.org/10.1111/nhs.12603	1.857	No	8	1	4	0	2	4	8	8
2	Downing, C. & Hastings-Tolsma, M., 2016, ‘An integrative review of Albertina Sisulu and Ubuntu: Relevance to caring and nursing’, *Health SA Gesondheid* 21, 214–227. https://doi.org/10.1016/j.hsag.2016.04.002	0.849	Yes	21	0	3	14	20	0	0	11
3	Downing, C., Hastings-Tolsma, M. & Nolte, A., 2016, ‘A critical evaluation on a Fulbright experience’, *Nursing Forum* 51(2), 117–124. https://doi.org/10.1111/nuf.12130	1.728	No	6	1	2	3	6	1	0	4
4	Myburgh, C., Poggenpoel, M. & Hastings-Tolsma, M., 2017, ‘Measuring dimensions of social climate among South African higher education students’, *Journal of Psychology in Africa* 27(6), 511–514. https://doi.org/10.1080/14330237.2017.1399552	0.93	No	7	0	5	6	7	0	0	7
5	Hastings-Tolsma, M., Nolte, A. & Temane, A., 2018, ‘Birth stories from South Africa: Voices unheard’, *Women and Birth: Journal of the Australian College of Midwives* 31(1), e42–e50. https://doi.org/10.1016/j.wombi.2017.06.015	3.172	No	22	1	18	20	22	1	20	9
6	Nolte, A., Downing, C, Temane, A. & Hastings-Tolsma, M., 2017, ‘Compassion fatigue in nurses: A metasynthesis’, *Journal of Clinical Nursing* 26, 23–24. https://doi.org/10.1111/jocn.13766	4.423	Yes	160	0	101	112	0	1	112	150
7	Nolte, A., Hastings-Tolsma, M. & Hoyte, F., 2015, ‘Midwifery management of asthma and allergies during pregnancy, birth, and the postpartum’, *British Journal of Midwifery* 23(4), 260–267. https://doi.org/10.12968/bjom.2015.23.4.260	0.292	No	4	0	0	3	4	0	0	1
8	Hastings-Tolsma, M. & Nolte, A.G., 2014, ‘Reconceptualising failure to rescue in midwifery: A concept analysis’, *Midwifery* 30(6), 585–594. https://doi.org/10.1016/j.midw.2014.02.005	2.64	Yes	8	0	0	8	8	1	0	8
9	Nolte, A., Hastings-Tolsma, M. & Harper, T., 2013, ‘Parturition and arthritis: A midwifery approach’, *British Journal of Midwifery* 21(8), 585–594. https://doi.org/10.1016/j.midw.2014.02.005	0.4	No	2	0	0	2	2	0	-	2
10	Hastings-Tolsma, M. & Nolte, A., 2013, ‘Two countries, one lens: Midwifery collaboration between the USA & the RSA’, *Sensitive Midwifery* 46–48.	None	Yes	2	0	0	0	2	0	-	1
11	Hastings-Tolsma, M., Hensley, J., Koschoreck, K., Patterson, E., Terada, M. & Bernard, R., 2012, ‘Nature and frequency of perineal hygiene and chorioamnioinitis’, in [Abstract] *10th Annual Congress of the Society of Midwives of South Africa*, Pretoria.	None	No	2	0	0	0	2	0	-	1
12	Downing, C., Temane, A., Bader, S.G., Hillyer, J.L., Beatty, S.C. & Hastings-Tolsma, M., 2021, ‘International nursing research collaboration: Visualizing the output and impact of a Fulbright Award’, *International Journal of Africa Nursing Sciences* 15, 100380. https://doi.org/10.1016/j.ijans.2021.100380	1.33	Yes	4	0	0	3	3	0	0	4
13	Hastings-Tolsma, M., Temane, A., Tagutanazvo, O.B., Lukhele, S. & Nolte, A.G., 2021, ‘Experience of midwives in providing care to labouring women in varied healthcare settings: A qualitative study’, *Health SA Gesondheid* 26, a1524. https://doi.org/10.4102/hsag.v26i0.1524	0.849	Yes	2	0	3	1	2	-	-	1
14	Nolte, A. & Downing, C., 2019, ‘Ubuntu-the essence of caring and being: A concept analysis’, *Holistic Nursing Practice* 33(1), 9–16. https://doi.org/10.1097/HNP.0000000000000302	1.02	No	21	0	8	9	2	0	-	16
15	Ntshingila, N., Downing, C. & Hastings-Tolsma, M., 2021, ‘A concept analysis of self-leadership: The “BleedingEdge” in nursing leadership’, *Nursing Forum* 56(2), 404–412. https://doi.org/10.1111/nuf.12551.	1.89	No	4	0	1	1	4	0	1	1
16	De Klerk, T., Temane, A. & Downing, C., 2023, ‘The development and implementation of a model to facilitate self-awareness of professionalism for enrolled nurses’, *Journal of Holistic Nursing*, epub ahead of print. https://doi.org/10.1177/08980101221134758	2.19	Yes	0	0	0	0	0	0	0	0

Note: Please see the full reference list of this article, https://doi.org/10.4102/hsag.v30i0.2776 for more information.

JIF, Journal Impact Factor; CINAHL, Cumulative Index to Nursing and Allied Health Literature.

#### Identification of co-citations

Sixteen primary studies were searched for citation usage from 01 January 2013 through 15 January 2023. PubMed MEDLINE was searched to verify all author names, article titles, journal titles and any other missing parts of the 16 published articles. Databases vary in the number of journals where articles are indexed, abstracted or published in full text, resulting in the need to search multiple databases. The fact that not all databases have a complete record of information regarding published works necessitated the need to conduct a wide search of the literature.

A total of seven databases and two alternative sources were searched to capture the articles citing the 16 published primary works. The seven databases reviewed were Scopus, Web of Science, PubMed, Embase, CINAHL (Cumulative Index to Nursing and Allied Health Literature), Papers First and Proceedings First. Alternative sources searched were Google Scholar and ResearchGate. ResearchGate produced 224 citations, far exceeding the numbers produced by academic databases.

Searching *Scopus* and the *Web of Science Core Collection* databases, author name variations for the 16 journal citations were searched, including author name misspellings that were identified. The search was conducted for all articles by each author during the search period confirming the author’s name. In addition, variations in title words reflecting British or American English word spellings were also searched. The search process below was followed:

- Last name I1
- Last name I1I2
- Last name I1I2I3
- Last name First name
- Last name First name I2I3
- Cited reference search.

*PubMed* was searched by focussing on author and journal name variations. The process produced citations that indicated which authors cited one of the 16 published articles and in which journal each article was published. The search process included all additional misspelled author or journal names that appeared in the Web of Science search process:

- Last name = Author family name as provided by the PI
- First name = Author given name as discovered through the database search or from the full text of the articles
- I1 = Author first initial
- I2 = Author second initial
- I3 = Author third initial

The cited reference search process was conducted using each article’s first author. If nothing was found, the search continued with the second author and then the remaining authors as appropriate.

*Embase* and *CINAHL* were searched using the same search procedure described for Scopus and Web of Science ensuring that no citations of the 16 original articles were missed. Results were found in both databases.

**FIGURE 1:**
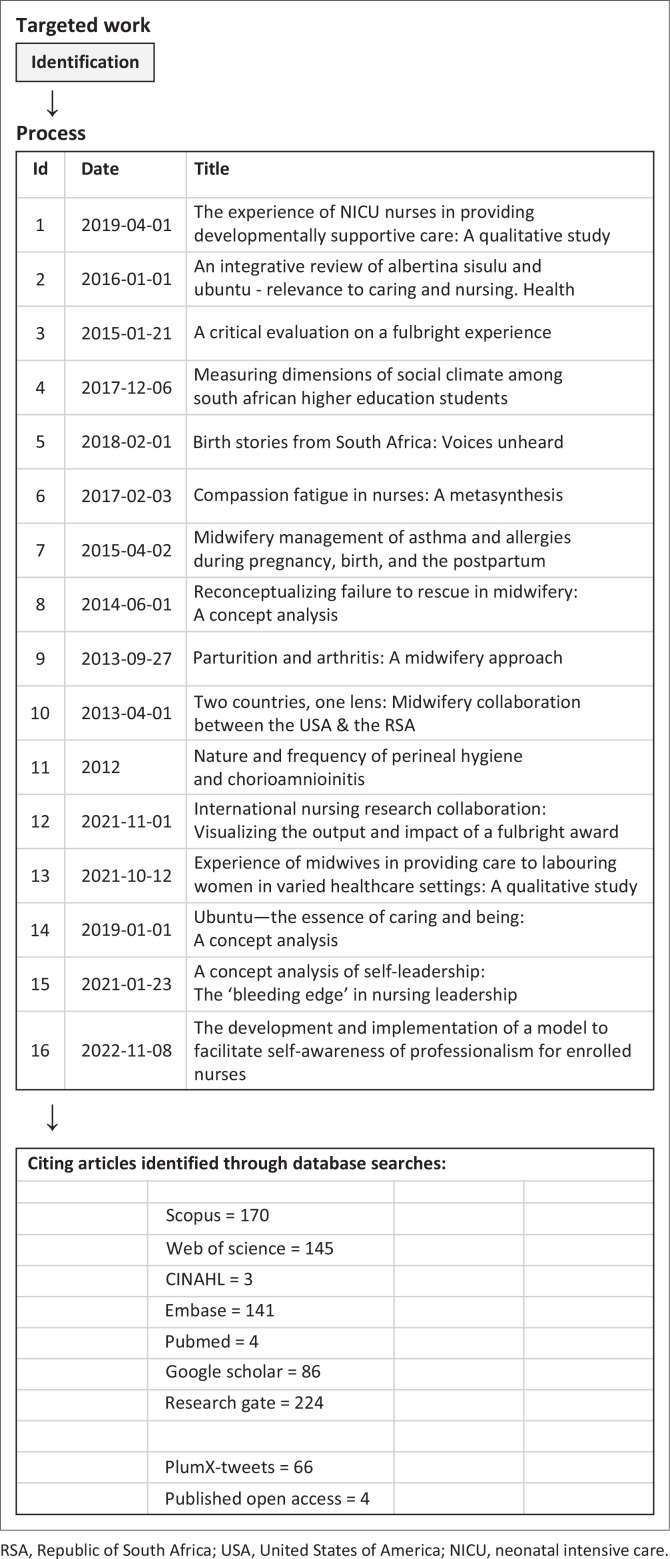
Search strategy and selection of articles related to the use of 16 primary articles.

*Google Scholar* and *ResearchGate* provided access to article citations and in some cases full-text articles, and both produced results. In both instances, author names and article titles were searched for all 16 original articles. Notably, ResearchGate produced the most results from all databases and alternative sources. This may be attributable to the ability of authors to self-index published articles. Presently, the breadth of the results provided a wider international scope than publisher-curated databases:

1. The search strategy for the selection of studies related to the use of the 16 primary works can be seen in Figure 1. All searching and identification of citing works was conducted by the librarian co-author.

#### Identification of non-scientific downstream citations

A noted gap in bibliometric literature is the examination of the downstream use of publications as noted through social media (Kokol & Blažun Vošner 2019). Such use can be determined using altmetrics or ‘alternative metrics’, which assist researchers in understanding how published works are being used and communicated to other end-users (Dardas et al. 2019).

To examine the use of each of the 16 primary published works in lay publications, grey literature, research blogs, mentions, reads and downloads, two altmetric services were utilised for analyses. These included PlumX Analytics and Altmetric, which were selected because the authors had access to academic libraries in possession of a licence for use. Such licence provided the altmetrics for the identified scholarly and creative works.

PlumX Analytics provided comprehensive item-level metrics, which provided insight into how individuals interacted with a given publication in an online environment. PlumX best collects Mendeley readership (i.e. users who have added an article to their library, demographic insights such as geographic information, discipline and academic status) (Ortega 2018). PlumX details five categories including citations, usage, captures, mentions and social media (Plum Analytics 2023).

Research has demonstrated that Altmetric.com provides the best coverage of blog posts, news and tweets (Ortega 2018). Altmetric required a DOI (digital object identifier) to generate an identified attention score – a whole number derived from an automated, weighted algorithm. This Altmetrics Attention Score (AAS) is an aggregated indicator that serves as a proxy for the volume and nature of attention – the downstream impact and in some cases public engagement, that a work receives online. The AAS begins to be generated immediately following publication and, in effect, serves as a real-time indicator of interest in the work (Lee, Choi & Michos 2021). Altmetric created a scoring system as a complement to traditional bibliometrics and tracked the online media presence of an article by measuring and compiling the mentions an article receives across varied outlets including Facebook, X (formerly Twitter), blogs, policy sources, Wikipedia and other platforms (Altmetric 2023). Mentions of a given article are weighted and a final Altmetric score reflects a summation of these weighted mentions.

#### Bibliometric Mapping

Bibliometric analysis here relied on Python and R (R Core Team 2020), two programming languages fundamental to modern academic data science, bibliometrics and statistical analysis. Their open-source communities have facilitated the development of numerous tools for data aggregation, mapping and visualisation. Data manipulation was applied using the ‘tidyverse’ package (Wickham, Vaughan & Girlich 2023), a collection of R packages designed for data science. For data visualisation, the authors employed ‘ggplot2’ (Wickham 2016). The authors incorporated network diagrams using the ‘igraph’ package (Csárdi et al. 2023). This package offers robust tools for network analysis and visualisation. Lastly, the authors utilised the Maps package (v3.3.0, ‘maps’ (Becker et al. 2018)) for generating geographical maps. This package provided a comprehensive set of map data useful in giving a geographic context to the academic influence and reach of the publications analysed.

### Ethical considerations

This research was reviewed on 16 March 2023, by the Baylor University Institutional Review Board and deemed exempt (IRBNet #2034964).

## Results

### Published works citing primary publications and growth trajectory

**FIGURE 2:**
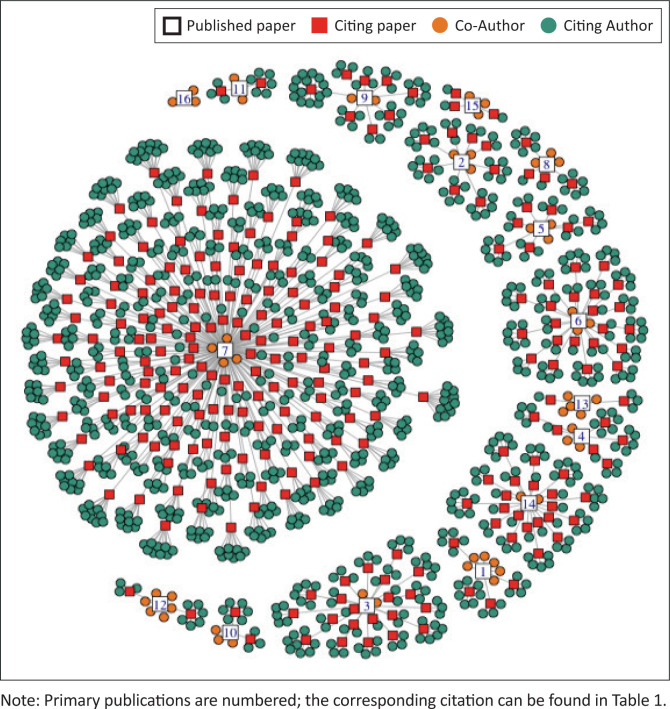
Citation counts (*n* = 273) resulting from primary works (*N* = 16), 2013–2023.

The 16 primary referenced works (see Table 1) were cited in 273 studies (see Figure 2). In the 2019 analysis of citations, primary studies (*N* = 11) had been cited 42 times (Downing et al. 2021). The citation receiving the greatest number of citations (*n* = 159) in the current analysis was the work related to compassion fatigue (Table 1, Paper 6) (Nolte et al. 2017). Five primary works received two or fewer citations (De Klerk, Temane & Downing 2023; Hastings-Tolsma et al. 2012, 2021; Hastings-Tolsma & Nolte 2013; Nolte, Hastings-Tolsma & Harper 2013). There was one primary work (Paper 16) that had no citations; 11 other citing publications (1, 3, 4, 7, 8, 9, 10, 11, 12, 13, 15) had eight or fewer secondary citations. Twelve (12) of the 16 primary publications had self-citations; four of the self-citations occurred within the first year following publication of the primary work.

### Impact of primary citations

To determine impact, cited primary works were examined for JIF, author-identified keywords, research methods employed by citing authors, focus of major clusters, disciplinary affiliation, geographic origin of first authors and presentations (see Table 2). In addition, non-scientific downstream of primary citations was examined to determine the influence on society (see Table 3).

**TABLE 2:** Citing studies (*n* = 273) resulting from primary works (*N* = 16).

Reference work*	Author (year)	Discipline(s)	Country of first author	Journal (impact factor)	Focus	Research design	Type publication
5	Adeagbo and Naidoo (2021)	Sociology	Johannesburg, South Africa	Journal of Adolescent Research (3.03)	Adolescent Parenting, Africa, Gender, Physical Health	Qualitative	Article
6	Ageel and Shbeer (2022)	Medicine	Jazan, Saudi Arabia	Journal of Healthcare Leadership (2.88)	Critical Care, Critical Care Unit, Nursing Practice, Quality of Life	Quantitative	Article
1	Albayrak and Büyükgönenç (2022)	Nursing	Istanbul, Turkey	Collegian (2.573)	Family-Centred Care, NICU	Quantitative	Article
6	Alharbi, Jackson and Usher (2019)	Health	Armidale, Australia	Nursing and Health Sciences (2.04)	Compassion Fatigue, Compassion Satisfaction, Critical Care Nurse, Resilience	Quantitative, Cross-Sectional	Article
6	Alkaissi et al. (2021)	Nursing	Nablus, Palestine	Research Square (not reported)	Nursing Education, Nursing Students, Resilience, Psychological Well-Being	Quantitative	Article
6	Alquwez et al. (2021)	Nursing	Al Dawadmi, Saudi Arabia	Nursing Open (1.942)	Caring Behaviour, Compassion Competence, Nursing Students, Self-Compassion	Quantitative	Article
6	Amsrud, Lyberg and Severinsson (2019)	Nursing	Kongsberg, Norway	Nurse Education in Practice (3.43)	Nurse education; Resilience development; Systematic review	Qualitative Systematic Review, Thematic Synthesis	Article
6	Arnold and Polifroni (2022)	Nursing	Connecticut, USA	(Book Chapter)	Complexity Science, Healthcare Delivery System,	Descriptive Essay	Book Chapter
8	Atsali and Russell (2018)	Nursing	Kenya, Africa	Africa Journal of Nursing and Midwifery (unknown)	Midwifery, Labour	Review	Article
6	Ayakdas et al. (2018)	Nursing	Izmir, Turkey	Medical Sciences (2.399)	Stress, Burnout	Quantitative, Descriptive	Article
6	Aydın, Kulakaç and Aydın Sayılan (2021)	Economics	Kirklareli, Turkey	International Journal of Clinical Practice (2.613)	Anxiety, Quality of Life, COVID-19, Healthcare Professionals	Quantitative	Article
6	Baguley et al. (2020)	Psychology	Auckland, New Zealand	Frontiers in Psychology (4.232)	Compassion, Emotion Regulation, healthcare, Physician-Patient Communication	Qualitative	Article
5	Bakker et al. (2021)	Medicine (obstetrics, gynaecology)	Malosa, Malawi	BMC Medical Ethics (3.917)	Informed Consent, Cesarean Birth	Qualitative	Article
6	Barone and Zakriževska-Belogrudova (2022)	Physiotherapists, Applied Sciences	Riga, Latvia	Proceedings of CBU in Social Sciences (not reported)	Burn Out, Compassion Fatigue, Secondary Traumatic Stress, Physiotherapy	Quantitative, Non-experimental	Article
6	Badwan, Eshah and Ahmad (2022)	Nursing	Zarqa, Jordan	Journal of Holistic Nursing and Midwifery (0.54)	Role, Perceived Organisational Support, Work Engagement, Intensive Care Nurse	Quantitative	Article
6	Baqeas, Davis and Copnell (2021)	Nursing, Midwifery	Bundoora, Australia	BMC Palliative Care (3.234)	Compassion Fatigue, Compassion Satisfaction, Palliative Care	Scoping Review	Article
6	Barone and Zakriževska-Belogrudova (2022)	Applied Sciences	Riga, Latvia	Proceedings of CBU in Social Sciences (unreported)	Burn out, Compassion fatigue, Secondary Traumatic Stress, Physiotherapy	Quantitative, Non-Experimental	Article
6	Barrué and Sánchez-Gómez (2021)	Nursing	Castellón, Spain	Enfermería Clínica (0.63)	Burnout, Compassion Satisfaction, Home Care Services, Hospice and Palliative Care Nursing, Job Satisfaction	Qualitative, Exploratory	Article
6	Bellali et al. (2019)	Nursing	Athens, Greece	Hellenic Journal of Nursing Science (not reported)	Emotional Labor, Healthcare Professionals, Secondary Traumatic Stress, Work Addiction	Quantitative	Article
6	Best et al. (2020)	Nursing	Bethesda, MD, USA	The Journal of Nurse Practitioners (0.767)	Compassion Fatigue, Military Practitioners, Mindfulness	Quantitative	Article
6	Bond et al. (2022)	Nursing, Midwifery	Sheffield, England, UK	International Journal of Mental Health Nursing (5.3)	Compassion, Discourse, Mental Health Nursing, Perspectives	Qualitative	Article
6	Bond et al. (2018)	Nursing	Derby, England, UK	Journal of Clinical Nursing (1.757)	Fatigue	Discourse Analysis	Article
6	Brodrick and Williamson (2020)	Midwifery, Psychology	London, England, UK	(Book)	Childbirth, Parturient	Descriptive Essay	Book Chapter
2	Burnell and Nel (2021)	Independent Scholar, Psychology	Bloomfontein, South Africa	Sociocultural Psychology of the Lifecourse book series (not reported)	Eugraphics, Sisulu, Ruth First, Anti-apartheid	Comparative Psychobiography	Article
6	Burrell et al. (2022)	Social Science	Arlington, VA, USA	International Journal of Smart Education and Urban Society (not reported)	U.S. Healthcare, COVID-19	Descriptive Essay	Article
6	Burrell et al. (2022)	Technology, Psychology	Melbourne, FL, USA	(Book)	Safety, U.S. Healthcare, COVID-19	Qualitative	Book Chapter
6	Buselli et al. (2020)	Occupational Health, Psychiatry	Cisanello (Pisa), Italy	Int J Environ Res Public Health (4.614)	COVID-19, Burnout, Compassion Satisfaction, Healthcare Workers, Professional Quality of Life, Secondary Traumatisation	Quantitative	Article
6	Butterworth et al. (2021)	Nursing	Watsonville, CA, USA	Journal of Nursing Administration (1.737)	Paediatrics, Cancer, Volunteering, Family Camp, Paediatric ICU Nurses	Quantitative	Article
3	Cagney, Pope and De Marrais (2018)	Education	Waterford, Ireland	Paper (no journal)	Fulbright, Collaboration, International, Research Partnerships	Qualitative, Descriptive	Article
6	Carleton et al. (2019)	Nursing	Regina, Saskatchewan, Canada	Canadian Journal of Behavioural Science (0.812)	Trauma	Quantitative	Article
5	Carlsson, Larsson and Jormfeldt (2020)	Midwifery	Halmstad, Sweden	International Journal of Qualitative Studies on Health and Well-being (1.947)	Childbirth, Place, Space	Qualitative, Critical Interpretive Synthesis	Article
2	Cahyani et al. (2021)	Nursing	Makassar, Indonesia	Enfermería Clínica (0.63)	Caring, Neoplasms, Watson’s theory	Qualitative, Descriptive	Article
6	Chen et al. (2022)	Nursing	Chengdu, China	Nursing Open (1.942)	Burnout, Compassion Fatigue, Compassion Satisfaction, Haematology, Cancer, Nurses	Quantitative, Cross-Sectional	Article
5	Cheruiyot and Brysiewicz (2019a)	Nursing/Midwifery	Durban, South Africa	International Journal of Africa Nursing Sciences (3.172)	Caring, Rehabilitation Nursing	Qualitative, Exploratory, Descriptive	Article
5	Cheruiyot and Brysiewicz (2019b)	Nursing/Midwifery	Durban, South Africa	Africa Journal of Nursing and Midwifery (0.25)	Inpatients, Rehabilitation nursing, Caring encounter, Uncaring encounter	Qualitative, Exploratory, Descriptive	Article
6	Chia-Yun, Chia-Chan and Ruey-Hsia (2021)	Nursing	Kaohsiung, Taiwan	Journal of Nursing Research (2.152)	Professional Nurses, Quality of Life, Physical Health, Mental Health, Compassion Fatigue	Quantitative	Article
14	Chowdhury et al. (2021)	Nursing	Malaysia	(Book)	Ubuntu Philosophy, Individualism	Qualitative	Book Chapter
14	Chowdhury et al. (2023)	Multidisciplinary	Malaysia	Ubuntu Philosophy for the New Normalcy (Book)	Ubuntu, Individualism, Features of Ubuntu	Descriptive Philosophical	Book Chapter
6	Clancy and Oyefeso (2019)	Nursing, Mental Health/Social Work, Health, Education	London, UK	Journal of Addictions Nursing (0.400)	Addiction	Mixed Methods	Article
6	Cook, Cai and Wohn (2022)	Information Systems	Taipei, Taiwan	Proceedings of the ACM on Human-Computer Interaction (0.617)	Content Moderation, Stress, Compassion Fatigue, Emotional Distress, Social Networking Sites	Quantitative	Article
6	Cross (2019)	Nursing	Danvers, MA, USA	Journal of Hospice and Palliative Nursing (0.708)	Compassion Fatigue	Qualitative	Article
5	Curtin et al. (2020)	Nursing/Midwifery	Cork and Dublin, Ireland	Journal of Clinical Nursing (4.423)	Birth, Concept Analysis, Humanisation, Labour, Pregnancy	Concept Analysis	Article
5	Curtin et al. (2022)	Midwifery	Dublin, Ireland	Women and Birth (3.172)	Labour, Parturition, Humanisation, Pregnancy High-Risk	Qualitative, Meta-Synthesis	Article
6	Dawood and Gamston (2019)	Nursing	London, UK	Emergency Nurse (unknown)	Recruitment, Retention	Quantitative	Article
7	De Gouveia Belinelo (2020)	Medicine	Callaghan, Australia	(Thesis)	Asthma in Pregnancy, Infant Lung Function, Breastfeeding, Birth Order	Quantitative	Thesis
6	Dicks et al. (2020)	Nursing	Canberra, Australia	Transplantation Direct (1.88)	Grief, Stress, Trauma, Organ Donation	Qualitative	Article
6	Dığın et al. (2022)	Nursing	Edime, Turkey	Online Turkish Journal of Health Sciences (unreported)	Perception of Quality of Care, Surgical nurse, Compassion fatigue	Quantitative	Article
6	Doleman and Duffield (2021)	Nursing	Perth, Australia	(Book Chapter)	Nursing Administration, Nursing Informatics	Descriptive Essay	Book Chapter
14	Dormehl (2021)	Nursing	Johannesburg, South Africa	(Thesis)	Assessment, Caring, Clinical Environment, Clinical Instructor, Perception, Student Nurse	Quantitative, Descriptive	Thesis
1, 7, 8, 9, 10, 11	Downing et al. (2021)	Nursing	Johannesburg, South Africa	International Journal of Africa Nursing Sciences (1.33)	Research Collaboration and Fulbright Award Impact	Quantitative, Bibliometric Mapping	Article
**2**	Downing, Poggenpoel and Myburgh (2017)	Nursing	Johannesburg, South Africa	Health SA Gesondheid (0.78)	Movement, Wholeness, Mental Health	Model Development, Qualitative	Article
**7, 8, 9, 10, 11**	Downing, Hastings-Tolsma and Nolte (2015)	Nursing, Midwifery	Johannesburg, South Africa	Nursing Forum (1.33)	Fulbright, Evaluation	Discourse, Evaluation	Article
2, 3, 5, 14	Downing et al. (2021)	Nursing, Midwifery	Johannesburg, South Africa	International Journal of Africa Nursing Sciences (1.728)	Fulbright Award, Research Collaborations,	Quantitative, Bibliometric Mapping	Article
5	Drysdale et al. (2021)	Human Development, Child Health	Johannesburg, South Africa	Maternal and Child Nutrition (3.092)	Breastfeeding, Depression, Fathers, Low Birth Weight, Maternal Mental Health, Maternal Public Health, Pregnancy	Quantitative	Article
5	Drysdale et al. (2022)	Human Development, Child Health	Johannesburg, South Africa	The South African Journal of Child Health (0.49)	Pregnancy, Male Partner Experiences, Ultrasound scans	Qualitative, Descriptive	Article
6	Dumit and Honein-AbouHaidar (2019)	Nursing	Beirut, Lebanon	Journal of Nursing Scholarship (2.500)	Fatigue, Depleted Compassionate Care	Qualitative	Article
14	Elsdörfer (2021)	Psychology, Anthropology	Kirkland, WA, USA	MAHABBAH: Journal of Religion and Education (0.47)	Western Psychology, Cultural Psychology, Healthcare Pandemics, churches, counselling, therapy, post-colonial settings	Descriptive Essay	Article
14	Elsdörfer (2021)	Pastoral Psychologist, Cultural Anthropology	Potchefstroom, South Africa	(Book)	Western psychology, Cultural psychology healthcare in pandemics, Churches, Counselling, Therapy Post-colonial settings	Descriptive Essay	Book Chapter
6	Fernández-Medina et al. (2021)	Nursing, Physiotherapy, Medicine	Almeria, Spain	Healthcare (Basel) (2.194)	Home Care, Vulnerable Patient	Qualitative	Article
6	Filipponi et al. (2022)	Medicine, Nursing (Oncology)	Milan, Italy	Psychological Reports (0.667)	Compassion Fatigue, Decision-Making, Emotion, Emotional Intelligence,	Quantitative	Article
6	Foli et al. (2020)	Nursing	West Lafayette, Indiana, USA	Archives of Psychiatric Nursing (2.02)	Content Analysis, Nurses, Psychological Trauma	Qualitative	Article
6	Foye, Simpson and Reynolds (2020)	Psychiatry, Psychology, Neuroscience	London, England, UK	Journal of Psychiatric and Mental Health Nursing (2.259)	Acute Care, Acute Hospital, Service Evaluation	Qualitative	Article
6	Fukumori et al. (2019)	Psycho-Oncology	Tokushima, Japan	Journal of Pain and Symptom Management (3.249)	Cancer, Compassion Fatigue, Nurses, Secondary Traumatic Stress, Traumatic Events	Quantitative	Article
6	Garrigues, Soulé and Vermeesch (2022)	Nursing	Portland, Oregon, USA	International Journal of Environmental Research and Public Health (3.39)	Nature, Connectivity, Natural Worlds, Attunement	Pedagogy Project	Article
6	Ghafourifard et al. (2022)	Nursing, Midwifery	Tabriz, Iran	Nursing Ethics (1.957)	Compassionate Care, Compassion, Nursing Care, Nursing Models	Qualitative, Grounded Theory	Article
6	Giesbrecht et al. (2021)	Nursing, Aging	Victoria, British Columbia, Canada	Social Science Medicine (5.379)	Emotional Geographies, End-of-Life Care, Healthcare Providers, Long-Term Care, Nursing Care, Residential Facilities	Qualitative	Article
14	Gilliam (2021)	Education	Los Angeles, CA, USA	(Thesis)	Advocating, Collaborating, Community, Education, Empowerment, Engagement, Social Capital	Qualitative	Thesis
6	Goktas, Gezginci and Kartal (2022)	Nursing	Istanbul, Turkey	Journal of Emergency Nursing (1.489)	Communication Skills, Compassion Fatigue, Emergency, Job Satisfaction, Motivation	Quantitative, Randomised Clinical Trial	Article
6	Golmenko et al. (2021)	Nursing	Irkutsk Oblast, Russia	Acta Biomedica Scientifica (1.35)	Burnout, Intervention, Management, Nurse	Literature Review	Article
6	Graves, Joyce and Hegazi (2022)	Chiropracter	Sydney, Australia	(Book Chapter)	Empathy, Compassion Fatigue, Health, Healthcare, Burnout, Secondary Traumatic Stress, Vicarious Trauma	Narrative Review	Book Chapter
6	Greinacher et al. (2021)	Internal Medicine, Psychosomatics	Heidelberg, Germany	Journal of Clinical Nursing (4.423)	Nursing Retention, Personality	Quantitative, Cross-Sectional	Article
1	Griffiths et al. (2021b)	Nursing	Sydney, Australia	Australian Critical Care (3.265)	Neonatal Developmental ICU Care	Quantitative	Article
1	Griffiths et al. (2021a)	Nursing	Westmead, Australia	Journal of Neonatal Nursing (0.9580)	NICU, Neurodevelopment	Integrative Review	Article
6	Gulliver et al. (2021)	Population Health, Mental Health	Canberra, Australia	Evaluation and Programme Planning (1.24)	Alzheimer’s Disease, Dementia, Depression, Music	Quantitative	Article
6	Günüşen et al. (2021)	Nursing	Izmir, Turkey	Perspectives in Psychiatric Care (2.186)	Burnout, Cognitive-Behavioural Approach, Compassion Fatigue, Nursing, Psychological Distress	Randomised Clinical Trial	Article
6	Gustafsson and Hemberg (2021)	Education, Welfare Studies	Vaasa, Finland	Nursing Ethics (3.344)	Compassion Fatigue, Experiences, Nurses	Qualitative	Article
6	Haqbin (2022)	Engineering	Latium, Italy	Unknown	Not known	Meta-synthesis	Article
14	Hardin (2015)	Nursing	Charlotte, NC, USA	Unknown	Not known	Descriptive Essay, Theory Development	Article
**14**	Hardin and Bishop (2013)	Nursing	San Antonio, TX, USA	(Book)	Academic Underachievement, Management, Vulnerable Learners, Critical Discourse Analysis	Qualitative	Book Chapter
6	Harris and Tao (2021)	Nursing	Orlando, Florida, USA	Journal of Religion and Health (2.732)	Religion, Spirituality, Mental Well-Being, Burnout, Nurses	Quantitative	Article
**10**	Hastings-Tolsma et al. (2013)	Midwifery, Nursing	Dallas, Texas, USA	Society of Midwives of South Africa 10^th^ Congress (N/A)	Labour Care, Infection	Quantitative	Conference Proceedings (abstract)
**6, 8**	Hastings-Tolsma et al. (2018)	Midwifery, Obstetrics and Gynaecology	Dallas, Texas, USA	Women and Birth (2.079)	Patient Birth Stories, Midwifery	Qualitative	Article
5, 8	Hastings-Tolsma et al. (2021)	Midwifery	Dallas, Texas, USA	Health SA Gesondheid (0.76)	Midwifery, Childbirth, Labour, Sub-Saharan Africa	Qualitative	Article
2	Hattingh and Downing (2020)	Nursing	Johannesburg, South Africa	International Journal of Africa Nursing Sciences (1.33)	Critical Care, Clinical Learning, Role Stress, Adaptation, Coping	Qualitative, Descriptive, Phenomenology	Article
6	Hidalgo-Andrade and Martínez-Rodríguez (2020)	Psychology	Quito, Ecuador	Interdisciplinaria (0.57)	Caregivers, Compassion Fatigue, Self-Care, Cognitive-Existential Psychology	Quantitative	Article
6	Hill (2021)	Nursing, Midwifery	Shreveport, LA, USA	Iranian Journal of Nursing and Midwifery Research (1.38)	Burnout, Compassion Fatigue, COVID-19, Post-traumatic Stress Disorder,	Descriptive Essay	Article
7	Hindley (2018)	Midwifery	Manchester, England, UK	British Journal of Midwifery (0.292)	Asthma, Pregnancy, PAAP, Emergency Care, National Guidelines	Descriptive Essay	Article
14	Hlojeng and Makura (2020)	Technology	Bloemfontein, South Africa	Proceedings of ADVED 2020- 6th International Conference on Advances in Education (no JIF)	Student Underachievement, Vulnerable Learners	Quantitative	Conference
2	Imafidon (2021)	Religion, Philosophy	London, England, UK	International Journal of Critical Diversity Studies (not reported)	African, Communitarian Philosophy, Ontology of Exclusion	Essay	Article
6	Iversen and Robertson (2021)	Neuroscience, Psychology	Leicester, England, UK	Psychiatry, Psychology and Law (0.744)	Attorney, Burnout, Compassion Fatigue, Lawyer, Legal Profession, Post-Traumatic Stress Disorder, Secondary Traumatic Stress, Solicitor	Review of Literature	Article
6	Jack et al. (2020)	Nursing	Hamilton, Ontario, Canada	Journal of Clinical Nursing (4.423)	Guidance, Intimate Partner Violence, Mobile Health, Quality Assurance, Telehealth	Discursive Paper	Article
6	Jakimowicz, Perry and Lewis (2017)	Nursing	Sidney, Australia	Journal of Clinical Nursing (1.757)	Nursing	Qualitative, Charmaz’s Grounded Theory	Article
14	Jansen-van Vuuren, Aldersey and Lysaght (2020)	Rehabilitation Therapy	Kingston, Ontario, Canada	Disability and Rehabilitation (2.054)	Africa, Occupational Therapy, Role, Scope, Culture, Advocacy	Qualitative	Article
14	Jansen-van Vuuren et al. (2021)	Rehabilitation Therapy	Kingston, Ontario, Canada	Quality of Life Research (4.147)	Africa, Children with Disabilities, Family Quality of Life	Scoping Review	Article
6	Jarrad et al. (2018)	Psychiatry	Amman, Jordan	Annals of General Psychiatry (2.158)	Compassion Fatigue	Quantitative, Descriptive Survey	Article
6	Jobe, Gillespie and Schwytzer (2021)	Nursing	Cincinnati, Ohio, USA	Journal of Trauma Nursing (0.64)	Trauma Nursing, Traumatic Stress Disorders, Compassion Fatigue	Quantitative	Article
15	Juurioksa (2022)	Health Promotion?	Johannesburg, South Africa	(Master’s Thesis)	Self-Management	Literature Review	Thesis
6	Kabunga et al. (2021)	Psychiatry	Lira, Uganda	PLoS ONE (3.752)	Compassion Fatigue, Nurses	Quantitative	Article
2	Keikelame and Swartz (2018)	Behavioural Sciences, Neurosciences and Neurology, Psychiatry	Cape Town, South Africa	Epilepsy and Behaviour (2.378)	Care	Qualitative	Article
6	Kelly (2020)	Nursing	Phoenix, AZ, USA	Critical Care Nursing Quarterly (1.57)	Burnout, Compassion Fatigue, Compassion Satisfaction, Secondary Trauma	Descriptive Essay	Article
2	Kobe, Downing and Poggenpoel (2020)	Nursing	Johannesburg, South Africa	Curationis (1.71)	Nursing Students, Caring	Qualitative	Article
6	Kraemer et al. (2022)	Nursing	Boston, MA, USA	(Book)	Healthcare Professionals, Burnout, Empathy Fatigue, Mindfulness, Compassion	Descriptive Essay	Book
6	Kreitzer, Brintnell and Austin (2019)	Social Work, Occupational Therapy, Nursing	Edmonton, Alberta, Canada	British Journal of Social Work (1.884)	Compassion Fatigue, Healthy Workplace	Qualitative	Article
15	Laiho and Olaste (2022)	Nursing	Tempere, Finland	(Master’s Thesis)	Self-Leadership, Burnout, Nurse, Supervisor, Organization	Systematic Review, Content Analysis	Thesis
5	Lappeman and Swartz (2022)	Psychology	Cape Town, South Africa	British Journal of Psychotherapy (0.49)	Milieu, Neglect, Obstetric Violence	Qualitative, Exploratory	Article
3	Lasater (2020)	Nursing	Portland, Oregon, USA	Nurse Educator (2.518)	Fulbright Program, Global Health, Nursing Education and Faculty, Transcultural Nursing	Descriptive Essay	Article
1	Lee, Park and Cho (2022)	Nursing	Iksan, South Korea	Child Health Nursing Research (0.72)	Premature Infant Growth and Development	Scoping Review	Article
6	Levkovich and Ricon (2020)	Education, Welfare Studies	Halfa, Israel	Asia Pacific Journal of Counselling and Psychotherapy (0.4)	School Counsellors, Compassion Fatigue, Secondary Trauma, Burnout, Emotional Distress	Quantitative	Article
6	Losung et al. (2021)	Applied Psychology	Melbourne, Australia	Journal of Police and Criminal Psychology (1.936)	Empathy, Professional Quality of Life, Sexual Assault, Child Abuse	Qualitative	Article
5	MacLellan (2022)	Nursing	Oxford, England	Health (London) (3.132)	Gender and Health, Maternity Care	Qualitative, Narrative Analysis	Article
13	Magqadiyane (2022)	Midwifery	Johannesburg, South Africa	(Thesis)	Physicians, Maternal Health Facilities, Maternal Health Care, Midwives, Quality of Care	Qualitative	Thesis
14	Mahilall (2021)	Education	Cape Town, South Africa	(Thesis)	Spiritual Care, Hospice, Palliative Care	Mixed Methods	Thesis
6	Maillet and Read (2021)	Administration, Nursing	Moncton, NB, Canada	Nursing Reports (0.8)	Compassion Fatigue, Compassion Satisfaction, Job Strain, Psychological Demands, Decision Latitude, Social Support, Emotional Intelligence, Nursing	Quantitative	Article
14	Muhammad-Lawal et al. (2022)	Nursing, Midwifery	Pretoria, South Africa	International Journal for Human Caring (0)	Philosophy, Caring, Student Attitudes Evaluation, Students, Nursing Psychosocial Factors, Humanism	Qualitative	Article
14	Mulaudzi et al. (2022)	Nursing	Pretoria, South Africa	Frontiers in Sociology (2.28)	Caregiving, Ubuntu Perspective, Restrictive Visitation Policy, COVID-19	Qualitative	Article
6	Mamdani et al. (2021)	Public Health	Vancouver, Canada	Substance Use and Misuse (2.164)	Compassion Fatigue, Burnout, Compassion Satisfaction, Overdose Prevention Sites, Overdose Related Deaths, Peer Responders, Secondary Traumatic Stress	Quantitative	Article
6	Mamdani et al. (2023)	Public Health	Vancouver, Canada	Substance Use and Misuse (2.164)	Compassion Fatigue, Burnout, Compassion Satisfaction, Overdose Prevention sites, Overdose related Deaths, Overdose Response	Quantitative	Article
5	Mapumulo et al. (2021)	Environmental and Rural Health	Durban, South Africa	PLoS ONE (3.752)	Disrespectful Care, Labour,	Qualitative, Longitudinal	Article
2	Marais and Coetsee (2020)	Political Science	Bloemfontein, Free State, South Africa	(Thesis)	Corporate Governance, Servant Leadership, Civil Society, Ubuntu, Ethical Leadership;	Qualitative	Thesis
6	Marshman, Hansen and Munro (2021)	Nursing, Midwifery	Victoria, Australia	Journal of Psychiatric and Mental Health Nursing (2.952)	Compassion Fatigue, Mental Health Nurse, Resilience, Self-Care,	Systematic Review	Article
15	Matahela (2021)	Nursing	South Africa	Journal of Nursing Education and Practice (0.2)	Leadership, Motivation, Nurse Educator, Nursing Education Institution, Reflection, Self-Leadership	Qualitative	Article
15	Matahela and Van Rensburg (2021)	Nursing	South Africa	Open Nursing (1.942)	Nurse Educators, Autonomy-Supportive Environment, Self-Leadership	Mixed Methods	Article
2	Mathebula, Downing and Kearns (2022)	Nursing	Johannesburg, South Africa	International Journal of Africa Nursing Sciences (1.33)	Newly Qualified Professional Nurses, Caring	Qualitative, Descriptive, Exploratory	Article
14	Matshaka, Downing and Poggenpoel (2020)	Nursing	Johannesburg, South Africa	Journal of Holistic Nursing (1.506)	Awareness, Caring, Context, Student Nurses	Quantitative, Descriptive	Article
2	Mayer, Von Niekerk and Fouché (2022)	Industrial Psychology and Management	Johannesburg, South Africa	Psychobiography	Sisulu, Ubuntu, Servant Leadership	Descriptive Essay	Book Chapter
6	Mento et al. (2021)	unknown	Sicily, Italy	Psychologia (0.28)	secondary traumatisation, compassion fatigue, vicarious trauma, burnout, healthcare professionals	Systematic Review	Article
6	Mercedes and Burrell (2020)	Psychology	Washington, DC, USA	PSU Research Review (1.9)	Business Sustainability, Strategic Change, Health Management, COVID-19, Health Administration, Management Adaptability	Quantitative, Case Study	Article
2	Michaelson, King and Pickett (2018)	Public, Environmental and Occupational Health, Pediatrics: Biomedical Social Sciences	Kinston, Ontario, Canada	Holistic Health in Children: Conceptualisation, Assessment and Potential	Holism	Discourse	Book Chapter
6	Missouridou et al. (2021a)	Nursing	Athens, Greece	Materia Socio-Medica (0.9)	Compassion Satisfaction, Compassion Fatigue, Emotional Work, Professional Quality of Life Scale, Secondary Traumatic Stress	Qualitative	Article
6	Missouridou et al. (2021b)	Nursing	Athens, Greece	Addictions Nursing (0.57)	Compassion Satisfaction, Emotional Work, Secondary Post-traumatic Stress	Mixed Methods	Article
5	Miyauchi, Shishido and Horiuchi (2022)	Nursing	Tokyo, Japan	Japan Journal of Nursing Science (1.85)	Childbirth, Maternal Health Services, Perception; Professional Patient Relations	Qualitative, Meta-synthesis	Article
14	Modic (2022)	Nursing	Cleveland, OH, USA	Nurse Leader (0.67)	Ubuntu, Magnus, Caregivers, Stress	Descriptive Essay	Article
12	Montegrico et al. (2022)	Nursing	Charlotte, NC, USA	Nurse Education Today (0.993)	COVID-19	Qualitative	Article
12	Montegrico et al. (2023)	Nursing	Charlotte, NC, USA	Nurse Education Today (0.993)	Research Collaboration during COVID-19 Pandemic	Qualitative	Article
1	Moreno-Jiménez et al. (2022)	Education, Psychology	Madrid, Spain	Current Psychology (4.297)	Healthcare Workers and ICU Stress	Quantitative	Article
2, 14	Mulaudzi et al. (2022a)	Nursing	Pretoria, Gauteng, South Africa	SAGE Open Nursing (1.47)	Ubuntu; barriers; enablers; nurses; nursing care; retired nurses	Qualitative, Exploratory	Article
3	Mulaudzi et al. (2022b)	Nursing	Pretoria, Gauteng, South Africa	African Journal of Development Studies (not reported)	Research Collaboration, Ubuntu	Narrative Case Study	Article
14	Muhammad-Lawal et al. (2022)	Nursing	Nigeria	International Journal for Human Caring (0.00)	Perceptions, Ubuntu, Caring, Nursing, Student Nurses	Qualitative	Article
5	Muthige, James and Morton (2019)	Midwifery	Elizabeth, RSA	British Journal of Midwifery (0.250)	Midwifery Role	Quantitative, Exploratory-Descriptive	Article
4	Myburgh, Poggenpoel and Fourie (2019)	Educational Psychology	Johannesburg, South Africa	Journal of Psychology in Africa (0.178)	Aggression	Quantitative	Article
4	Myburgh, Poggenpoel and Fourie (2020)	Educational Psychology	Johannesburg, South Africa	Curationis (1.71)	Aggression	Quantitative	Article
4	Myburgh, Poggenpoel and Fourie (2021)	Educational Psychology	Johannesburg, South Africa	Health SA Gesondheid (0.76)	Aggression	Quantitative	Article
2	Nash-Patel et al. (2022)	Nursing	London, England	Nurse Education Today (0.993)	Intellectual Learning Disability, Relational Learning, Social Justice	Qualitative	Article
2	Nash-Patel et al. (2023)	Nursing	London, England	Journal of Nursing Education (1.726)	Adolescent Learning Disabilities, Nursing Students	Qualitative	Article
6	Ndlovu et al. (2022)	Nursing	Pretoria, South Africa	Southern African Journal of Critical Care (0.24)	COVID-19, Compassion Fatigue; Compassion Satisfaction; Critical Care; Nurses; Professional Quality of Life	Quantitative, Cross-Sectional	Article
4	Ndoro and Martins (2019)	Educational Psychology	Grahamstown, South Africa	Journal of Psychology in Africa (0.178)	Employee Engagement, Higher Education	Quantitative, Survey	Article
6	Nègre et al. (2021)	Unknown	Gif-sur-Yvette, Frane	Douleurs (0.08)	COVID epidemic	Quantitative, Descriptive	Article
12	Nguyen et al. (2022)	Information Management	Wellington, New Zealand	Quantitative Science Studies (3.7)	International Research Collaboration	Quantitative Measurement Tool	Article
6	Nkabinde-Thamae (2021)	Nursing	Johannesburg, South Africa	(Dissertation)	Self-Care, Professional Nurses, Primary Care	Qualitative, Exploratory, Descriptive	Dissertation
2, 5	Nkabinde-Thamae, Downing and Nene (2022)	Nursing	Johannesburg, South Africa	Nursing Forum (0.14)	Mindfulness, Self-care, well-being	Qualitative	Article
6	Nkoane and Mavhandu-Mudzusi (2020)	Nursing	Tshwane, South Africa	Africa Journal of Nursing and Midwifery (0.25)	Community Service Nurses, Healthcare Services, District Public Hospital, South African Nursing Council	Qualitative	Article
**2**	Nolte and Downing (2017)	Nursing	New York, USA	Global Advances in Human Caring Literacy (N/A)	Human Caring	Discourse	Book Chapter
**2**	Nolte and Downing (2019)	Nursing/Midwifery, Integrative and Complementary Medicine	Johannesburg, South Africa	Holistic Nursing Practice (1.02)	Caring, Ubuntu	Concept Analysis	Article
6	O’Callaghan et al. (2019)	Nursing/Midwifery	Clayton, VIC, Australia	International Emergency Nursing (0.71)	Compassion Fatigue	Quantitative	Article
6	Ogino et al. (2019)	Nursing, Anesthesiology	Maebashi, Japan	Frontiers in Human Neuroscience (3.209)	Compassion Fatigue, Emotion-Demanding Profession	Quantitative	Article
6	Okoli et al. (2019)	Nursing, Social Work	Lexington, KY, USA	International Journal of Mental Health Nursing (5.1)	Burnout, Compassion Satisfaction, Healthcare Professionals	Quantitative	Article
2	Omodan (2022)	Education	Eastern Cape, South Africa	Journal of Ethnic and Cultural Studies (3.123)	Ubuntu, politics, conflict management,	Theoretical Discourse	Article
6	Ondrejková and Halamová (2022)	Applied Psychology	Bratislava, Slovakia	International Journal of Nursing Sciences (2.618)	Compassion Fatigue, Coping, Self-care, Nurses, Psychological Adaptation	Quantitative	Article
6	Osei et al. (2019)	Nursing	Silang, Cavite, Phillipines	Abstract Proceedings International Scholars Conference (not applicable)	Resilience, Compassion Fatigue, Registered Nurses	Quantitative	Conference Abstract
6	Ottoboni et al. (2021)	Psychology	Bologna, Italy	Dementia (2.624)	Education, Health Services, Personal Support, Psychosocial Care, Young Onset Dementia	Qualitative	Article
6	Owens et al. (2020)	Nursing, Medicine	Cleveland, Ohio, USA	Holistic Nursing Practice (1.02)	Mindfulness, Nurses’ Compassion Fatigue	Quantitative	Article
6	Pang et al. (2020)	Nursing	Seoul, Korea	International Nursing Review (2.871)	Burnout, Compassion Satisfaction, Depressive Symptoms, Professional Quality of Life, Secondary Traumatic Stress, Turnover Intention	Quantitative	Article
8	Patterson, Skinner and Foureur (2015)	Midwifery	Dunedin, New Zealand	Midwifery (2.048)	Decision-making, Transfer, Slow Labour	Qualitative, Thematic Analysis	Article
6	Pedersen and Obling (2019)	Business	Frederiksberg, Denmark	Sociology of Health and Illness (2.122)	Compassion	Policy Paper	Article
6	Pehlivan and Güner (2018)	Psychiatric Nursing	Istanbul, Turkey	Journal of Psychiatric Nursing (1.91)	Compassion Fatigue	Review	Article
6	Penz and Tipper (2019)	Nursing	Regina, SK, Canada	(Book)	Caregiver, Professional Quality of Life, Palliative Care, Hospice	Descriptive Essay	Book Chapter
6	Pérez-García et al. (2020)	Nursing	Huelva, Spain	International Journal of Mental Health Nursing (4.865)	Burnout, Compassion Fatigue, Post-Traumatic Stress Disorders	Qualitative	Article
6	Peters (2018)	Nursing	Tennessee, USA	Nursing Forum (1.33)	Nurses	Concept Analysis	Article
6	Plange-Kaye (2019)	Health Administration	Phoenix, AZ, USA	(Dissertation)	Barriers Care, Resources Care, Formal Caregiver	Qualitative, Phenomenology	Dissertation
3	Pope et al. (2017)	Adult Education	Carrollton, Georgia, USA	Learning Communities Journal (not reported)	Higher Education, Fulbright Award	Qualitative, Case Study	Article
14	Qamar (2022)	Science and Technology	Scania, Sweden	Journal of Early Childhood Research (1.45)	Value of the Child, Social Value, Global South, Social Construction Sociology of Childhood	Descriptive Essay	Article
6	Rabin et al. (2019)	Nursing	Porto Alegre, Brazil	Rev Esc Enferm USP (0.81)	Oncology Service, Hospital, Nursing Care, Oncology Nursing, Quality of Health Care, Patient Safety	Quantitative, Cross-Sectional	Article
6	Rajeswari et al. (2020)	Nursing	Andhra Pradesh, India	Iranian Journal of Nursing and Midwifery Research (1.38)	Burnout, Compassion Fatigue, Nurses	Quantitative	Article
6	Raustøl and Tveit (2022)	Nursing	Oslo, Norway	Nursing Ethics (3.344)	Compassion, Emotions, Nursing Education, Professional Ethics, Professional Formation	Theoretical Essay	Article
6	Rees et al. (2021)	Emergency Medical Technician	Wales, UK	British Paramedic Journal (0.6)	Ambulance, EMS, Heroism, Paramedic	Meta-synthesis	Article
6	Robertson, England and Khodabakhshi (2020)	Radiology	London, England, UK	Journal of Medical Imaging and Radiation Sciences (1.481)	Burnout, Compassion Fatigue, Empathy, Psychological Support, Radiography	Quantitative	Article
6	Robertson et al. (2022)	Radiography	London, England	Radiography (29.146)	Compassion Fatigue, Occupational Stress, Burnout, Well-being, Radiography	Systematic Review	Article
6	Rohner et al. (2022)	Psychology	Zurich, Switzerland	Frontiers in Psychology (4.232)	Adverse Childhood Experiences, Altruism Born of Suffering, Framework Analysis, Prosocial Behavior, Resilience	Qualitative	Article
6	Rouleau et al. (2019)	Nursing	Quebec, Canada	Journal of the Association of Nurses in AIDS Care (1.318)	Compassion	Qualitative, Exploratory	Article
5	Rucell (2019)	Health Policy, Sociology	Oxfordshire, England	(Book Chapter)	Obstetrical Structural Violence, Childbirth, Negligence Case Law	Essay	Book Chapter
6	Ruiz-Fernández, Pérez-García and Ortega-Galán (2020)	Nursing	Almeria, Spain	International Journal of Environmental Research and Public Health (4.615)	Compassion Fatigue, Compassion Satisfaction, Burnout, Socio-Demographic Factors, Work Related Factors	Quantitative	Article
6	Ruiz-Fernández et al. (2020)	Nursing	Almeria, Spain	Journal of Clinical Nursing (4.423)	COVID-19, burnout, Compassion Fatigue, Compassion Satisfaction,	Quantitative	Article
6	Ruiz-Fernández et al. (2021)	Nursing	Almeria, Spain	Research in Nursing and Health (2.228)	Empathy, Hospital/Institutional Environment, Job Related Stress, nursing care/interventions, Work Satisfaction	Quantitative	Article
6	Ryu and Shim (2022)	Nursing	Gyeongju, South Korea	Iranian Journal of Public Health (1.291)	Compassion Fatigue, Compassion Satisfaction, Patient Safety	Quantitative, Cross-Sectional	Article
6	Sabanciogullari, Yilmaz and Karabey (2021)	Nursing	Sivas, Turkey	Contemporary Nurse (1.49)	Compassion, Compassion Fatigue, Medical Error, Nurse	Quantitative	Article
6	Sabery and Sabery (2020)	Medical Sciences, Nursing	Kashan, Iran	Europe PMC (2.478)	Compassion Fatigue, Nursing	Qualitative	Article
6	Sadanandan et al. (2021)	Nursing	Vadodara, India	Journal of Pharmaceutical Research International (0.036)	Compassion Fatigue, Burnout, Compassion Satisfaction, Nurses Casualty, ICU	Quantitative	Article
6	Saghafi et al. (2021)	Nursing	Tehran, Iran	Trauma Monthly (0.39)	Job stress, Occupational Stress, Military Nurses	Systematic Review	Article
3	Salman (2019)	Nursing	Pennsylvania, USA	Journal of Professional Nursing (1.829)	Fulbright Experience	Discourse Analysis	Article
6	Salmond et al. (2019)	Nursing	Newark, New Jersey, USA	JBI Database of Systematic Reviews and Implementation Reports (0.74)	Compassion Fatigue	Review	Article
6	Sansó et al. (2020)	Midwifery, Physiotherapy	Palma de Mallorca, Spain	Int J Environ Res Public Health (4.614)	Self-are, Occupational Health, Burnout, Quality of Life,	Quantitative	Article
6	Sarabia-Cobo et al. (2021)	Nursing	Cantabria, Spain	International Journal of Environmental Research and Public Health (3.39)	Compassion Fatigue, Geriatric Nursing, Mental Health, Psychological, Stress	Quantitative	Article
7	Satria et al. (2022)	Medicine	Yogyakarta, Indonesia	Malaysian Journal of Medicine and Health Sciences (0.3)	Primary Health Care, Allergy Training, Knowledge Enhancement	Quantitative, Cross-Sectional	Article
6	Settineri et al. (2019)	Psychology	Messina, Italy	Journal of Mind and Medical Sciences (0.150)	Caregiver, Compassion Fatigue	Quantitative	Article
6	Schneider, Smith and Howard (2022)	Nursing	Somerville, New Jersey, USA	Nursing (2.577)	Burnout, Professional Prevention and Control, COVID-19, Humans Nursing Staff, Hospital, SARS-CoV-2, Workforce	Descriptive Essay	Article
6	Sciepura and Linos (2022)	Public Policy	Berkley, CA, USA	Review of Public Personnel Administration (4.072)	Employee Burnout, Compassion Fatigue, Social Belonging, Street-Level Bureaucrats, COVID-19	Quantitative	Article
6	Sebrant and Jong (2020)	Nursing	Nyköping, Sweden	Scandinavian Journal of Caring Sciences (2.32)	Caring, Nursing, Philosophy, Nursing Theory	Qualitative, Meta-Synthesis	Article
6	Shahar, Asher and Natan (2019)	Nursing	Netanya, Israel	Nursing and Health Sciences (1.321)	Compassion Fatigue	Quantitative	Article
1	Shin and Bang (2021)	Nursing	Seoul, South Korea	The Journal of Korean Academic Society of Nursing Education (0.984)	ICU, Premature Neonates, Growth and Development	Quantitative	Article
14	Siraz Chowdhury et al. (2023)	Unknown	Kuala Lumpur, Malaysia	(Book Chapter)	Ubuntu, Individualism, Features of Ubuntu	Descriptive Essay	Book Chapter
6	Shitaya, Nakamura and Sato (2018)	Nursing	Chiba, Japan	International Journal of Nursing Practice (1.189)	Nursing	Qualitative Descriptive	Article
6	Signal, Casey and Taylor (2022)	Psychology	Queensland, Australia	Traumatology (2.18)	Quality of Life, Compassion Fatigue, Animal Rescue	Qualitative	Article
6	Solomon (2021)	Nursing	Grenada	British Journal of Nursing (0.71)	COVID-19 pandemic, Nurse Volunteers, Nursing Theories	Descriptive Essay	Article
1	Song et al. (2021)	Nursing	Hefei, China	Japan Journal of Nursing Science (1.85)	Nursing Supportive Care, Cancer Patients	Quantitative	Article
**2**	Spies et al. (2017)	Nursing, Midwifery	Texas, USA	International Nursing Review (1.562)	Global Partnerships, Model Development	Discourse	Article
6	Stajduhar et al. (2020)	Nursing	Victoria, British Columbia, Canada	Palliative and Supportive Care (2.4)	Health Disparities, Marginalised Populations, Palliative Approach, Structurally Vulnerable Populations	Qualitative, Critical Ethnography	Article
6	Steinheiser, Crist and Shea (2020)	Nursing	Norwood, MA, USA	Applied Nursing Research (2.257)	Compassion Fatigue, Registered Nurses, Skilled Nursing	Qualitative	Article
6	Stevenson, Munro and Barrington (2020)	Psychology	London, England, UK	British Journal of Healthcare Management (0.54)	Oncology Nursing, Education, Psychosocial	Literature Review	Article
6	Straughair, Clarke and Machin (2019)	Nursing/Midwifery	Newcastle upon Tyne, UK	Journal of Advanced Nursing (2.267)	Compassion	Qualitative, Constructivist Grounded Theory	Article
12	Su, Hwang and Chang (2022)	Dentistry	Taipei City, Taiwan	Journal of Nursing Management (3.325)	Nursing Core Competencies	Quantitative, Bibliometric Mapping	Article
8	Symon et al. (2016)	Midwifery	Dundee, UK	BMC Pregnancy and Childbirth (2.413)	Pregnancy, Maternity Care, Midwifery Led	Systematic Review	Article
14	Tawiah (2020)	Education	Pretoria, South Africa	(Thesis)	Adult and Community Education and Training, Adult Education, Skills Training, Socio-Economic Development, Poverty, Rural Women	Qualitative	Thesis
6	Teixeira (2021)	Nursing	Rhode Island, USA	(Thesis)	Compassion Fatigue, Critical Care Nurses,	Quantitative	Thesis
6	Thapa et al. (2021)	Health, Medicine	Sydney, Australia	Nursing and Health Sciences (2.039)	Burnout, Compassion Fatigue, Resilience Healthcare Professionals	Editorial	Article
6	Theofilou et al. (2022a)	Health Sciences, Nursing	Athens, Greece	Journal of Public Health and Epidemiology (2.4)	Emergency Department, Fatigue, Greek Hospitals, Nursing Staff, Social Support	Quantitative, Cross-Sectional	Article
6	Theofilou, Iona and Tsironi (2022)	Social Sciences	Athens, Greece	World Journal of Clinical Medicine Research (not reported)	General Health, Fatigue, Social Support, Health Professionals	Quantitative	Article
6	Theofilou, Iona and Tzavella (2022b)	Social Sciences	Athens, Greece	Journal of Community Medicine and Health Care (1.9)	General Health, Fatigue, Social Support, Health Professionals	Quantitative	Article
14	Thinane (2022)	Humanities	Bloemfontein, South Africa	HTS Theological Studies (0.387)	Missio Dei, Missio Hominum, Ubuntu	Descriptive Essay	Article
6	Toebe et al. (2023)	Business, Management	Porto Alegre, Brazil	Revista Foco (not reported)	(not identified)	Quantitative	Article
6	Tokac and Razon (2021)	Nursing	St. Louis, Missouri, USA	Journal of Nursing Management (3.325)	COVID-19, Anxiety, Depression, Nurses, Work Impairment	Quantitative	Article
5	Turan, Suveren and Vural (2022)	Nursing, Midwifery	Serdivan, Turkey	Women and Health (1.377)	Childbirth Experiences, Maternal Care, Midwife	Qualitative	Article
6	Üstün and Dogan (2022)	Nursing	Amasya, Turkey	International Journal of Caring Sciences (1.11)	Compassion Fatigue, COVID-19, Emotional Labor, Job Burnout, Nurse, Secondary Trauma	Quantitative, Descriptive Correlational	Article
4	Valenzuela-García et al. (2022)	Educational Psychology	Sonora, Mexico	PsyEcology (1.31)	Positive School Environment, Sustainable Behaviour, Higher Education	Quantitative	Article
2	Van Der Westhuizen et al. (2020)	Radiography	Johannesburg, South Africa	Health SA Gesondheid (0.76)	Caring, Sonography	Qualitative, Phenomenology	Article
6	Vanobberghen et al. (2020)	Health Policy	Brussels, Belgium	BMC International Health and Human Rights (2.46)	Hunger Strike, Undocumented Migrants, Ethical Dilemmas, Secondary Traumatic Stress	Qualitative	Article
6	Van Overmeire et al. (2021)	Public Health	Brussels, Belgium	Journal of Public Health (Oxford) (2.341)	Mental Health, Screening	Quantitative	Article
2	Van Rensburg, Maree and Casteleihn (2017)	Oncology Nursing	Johannesburg, South Africa	Asia Pacific Journal of Oncology Nursing (1.539)	Cancer, Quality of Life	Qualitative, Exploratory	Article
14	Van Vuuren, Okyere and Aldersey (2020)	Occupational Therapy	Queensland, Australia	South African Journal of Occupational Therapy (1.1)	Occupational Therapy, Ubuntu	Scoping Review	Article
**4**	Van Zyl and Dhurup (2018)	Psychology	Vanderbijlpark, South Africa	Journal of Psychology in Africa (0.178)	University Students, Self-Efficacy, Satisfaction with Life, Happiness	Quantitative, Regression Analysis	Article
6	Vivolo, Owen and Fisher (2022)	Clinical Psychology	Norwich, England, UK	Mental Health and Prevention (2.69)	Burnout, Stress, Mental Health, Psychological Therapists, Psychologists	Qualitative Systematic Review, Meta-synthesis	Article
5	Wanyenze et al. (2022)	Nursing	Mbarara, Uganda	BMC Pregnancy and Childbirth (3.105)	Continuous Support, Birth Companion	Qualitative	Article
6	Walker and Efstathiou (2020)	Nursing	Wolverhampton, England, UK	Nursing in Critical Care (2.897)	Compassion Fatigue, Critical Care Nursing, Grief, ICU, Self-Care, Terminal Care	Discourse Essay	Article
13	Wibbelink, James and Thomson (2022)	Midwifery	Johannesburg, South Africa	African Journal of Midwifery and Women’s Health (0.316)	Midwifery Practice, Public Maternity Units, Public Hospitals	Qualitative	Article
6	Wentzel, Collins and Brylewicz (2019)	Nursing	Durban, South Africa	Health SA Gesondheid (1.14)	Compassion Fatigue	Qualitative	Article
6	Willems et al. (2020)	Psychology, Health Psychology	Rotterdam, Netherlands	International Journal of Environmental Research and Public Health (4.614)	Crisis Line, Volunteer, Mental Well-being, Influencing Factors,	Systematic Review	Article
6	Wu (2020)	Public Administration, Management	Tainan, Taiwan	Current Psychology (4.297)	Animal Protection, Compassion Fatigue, Mindfulness, Moral Disengagement	Qualitative	Article
6	Yakar et al. (2023)	Nursing	Istanbul, Turkey	International Journal of Occupational Safety and Ergonomics (2.54)	Caring, Chronic Disease, Compassion, Compassion Fatigue, Nurses	Quantitative, Descriptive	Article
6	Younas and Rasheed (2018)	Nursing	Newfoundland, Canada	Creative Nursing (0.12)	Compassion	Quantitative, Descriptive	Article
6	Yu, Qiao and Gui (2021)	Nursing	Shanghai, China	International Emergency Nursing (2.142)	Burnout, Compassion Fatigue, Compassion Satisfaction, Emergency Nurses, Empathy, Job Satisfaction, Self-Compassion	Quantitative, Cross-Sectional	Article
6	Zhang et al. (2018)	Neurology, Nursing, Urology, Respiratory Care	Jining, China	Medicine (0.190)	Compassion Fatigue, Burnout, Compassion Satisfaction	Systematic Review, Meta-analyses	Article
6	Zhang et al. (2021)	Nursing	Changsha, China	Journal of Advanced Nursing (3.187)	Burnout, Compassion Fatigue, Healthy Lifestyle, Nurses, Resilience, Self-Efficacy	Quantitative	Article
8	Zielinski, Brody and Low (2016)	Midwifery	Ann Arbor, Michigan, USA	JOGNN (1.219)	Physiologic Birth, Labor Support, Midwifery Model of Care	Discourse	Article
6	Zolkefli (2021)	Nursing	Bandar Seri Begawan, Brunei	International Journal of Care Scholars (not reported)	Nurse-Patient, Relationship, Difficult, Nursing	Descriptive Essay	Article

Note: Bolded numbers indicate self-citations. Please see the full reference list of this article, https://doi.org/10.4102/hsag.v30i0.2776 for more information.

N/A, not applicable; NICU, neonatal intensive care; ICU, intensive care unit; JOGNN, Journal of Obstetric, Gynecologic, & Neonatal Nursing; USA, United states of America; UK, United Kingdom.

* , Numbers correspond to numbered referenced primary works in Table 1.

**TABLE 3:** Downstream citations in non-scientific sources for primary sources (*N* = 16) using altmetrics.

Primary source	Usage			Captures		Citations	Social media		Attention score*
	Abstract views	Link-outs	Full-text views	Export saves	Readers	Citation indexes	Facebook (likes, shares and comments)	PlumXTweets	
Austin, Downing and Hastings-Tolsma (2019)	171	47	-	42	14	0	0	0	0
De Klerk et al. (2023)	-	-	-	-	-	-	-	-	-
Downing and Hastings-Tolsma (2016)	961	166	1,724	26	46	4	0	3	-
Downing, Hastings-Tolsma and Nolte (2016)	160	42	7	25	17	1	0	0	0
Downing et al. (2021)	-	-	-	-	-	-	-	-	-
Hastings-Tolsma et al. (2012)	-	-	-	-	-	-	-	-	-
Hastings-Tolsma and Nolte (2013)	Article appeared in non-indexed publication								
Hastings-Tolsma and Nolte (2014)	375	72		115	38	6	23	31	20
Hastings-Tolsma et al. (2018)	256	42	0	96	111	3	0	28	0
Hastings-Tolsma et al. (2021)	-	-	-	-	-	-	-	-	-
**Myburgh, Poggenpoel and Hastings-Tolsma (2017)	-	-	65	-	-	3	-	-	0
Nolte and Downing (2019)	-	-	-	-	-	-	-	-	-
Nolte et al. (2017)	2,592	1,717	6	333	143	27	0	32	19
Nolte, Hastings-Tolsma and Hoyte (2015)	415	47	74	34	2	1	-	-	0
Nolte et al. (2013)	338	18	61	39	10	1	0	0	0
Ntshingila, Downing and Hastings-Tolsma (2021)	-	-	-	-	-	-	-	-	-
Hastings-Tolsma et al. (2012)	-	-	-	-	-	-	-	-	-

Note: Citations, traditional citation indexes as well as citations indicative of societal impact (e.g. clinical or policy citations). Usage, number of clicks, downloads, views, library holdings and video plays. Captures, leading indicator of future citations and includes bookmarks, code forks, favourites, readers and watchers. Indicates that an individual wants to come back to work. Mentions, indicative of how people are really engaging in the research or article and include activities such as news articles and blog posts. Social media, indicative of the buzz and attention an article is receiving. It includes tweets, Facebook likes, shares and comments. Please see the full reference list of this article, https://doi.org/10.4102/hsag.v30i0.2776 for more information.

* , Attention Score is a feature of Altmetric indicating attention the work has received using a weighted count;

** , Journal metrics (views and crossref citations).

### Journal impact factor

The initial 2019 list of primary articles (*N* = 11) was referenced in journals with JIFs ranging from 0.0250 to 2.079. The current comprehensive assessment of primary articles (*N* = 16) demonstrated JIFs ranging from unreported to JIFs of 0.08 to 5.379. Specifically, an examination of the JIFs of citations emanating from the primary referenced works (*N* = 16) found that the lowest impact factor was 0.08 in *Douleurs* – a peer-reviewed journal that serves a multidisciplinary audience of professionals involved in the management of pain. The highest JIF was 5.379 in *Social Science & Medicine*, which provides an international and interdisciplinary forum for the dissemination of social science research on health.

Journals with a JIF ranging from four to five were distributed in the following range: lowest: 4.072 (*Review of Public Personnel Administration*) to 4.865 (*International Journal of Mental Health Nursing*). The JIF of 4.423 occurred on seven occasions and belonged to the *Journal of Clinical Nursing*. Twenty-seven articles were published in a journal with a JIF ranging from 3.928 to 3.03. In this range, three articles were published in *Women and Birth* (JIF 3.172), two in the *International Journal of Africa Nursing Sciences* (JIF 3.172), two articles in *Nursing Ethics* (JIF 3.344) and three articles in the *Journal of Nursing Management* (JIF 3.325).

### Keywords

In a previous study (Downing et al. 2021), the top author keywords were health (15), nurse (11), fatigue (10) and compassion (10), and these were considered the basic elements of the research niche or domain. In the current study, the general keywords demonstrated a broad overview of health and nursing and were identified as important research themes for this bibliometric analysis. High-frequency keywords were compassion fatigue (66), burnout (40), caring (11), Ubuntu (10), mental health and well-being (9) and mindfulness (6).

For the top cited studies, the keywords emanating from Paper 7 included compassion fatigue, burnout, vicarious trauma, traumatic stress, compassion satisfaction, compassionate care, coping and resilience. Twelve of the 160 studies identified the coronavirus disease 2019 (COVID-19) as a keyword. Paper 6 had 22 citations and common keywords included labour, childbirth, humanisation, caring and/or uncaring encounters, birth companion, obstetric violence and neglect. Papers 3 and 16 focussed on the theme of Ubuntu and common keywords were caring and/or caregiving, Ubuntu, nursing and/or healthcare professionals, community, quality of life and individualism.

### Focus major clusters

Studies citing one of the primary referenced studies (*N* = 16) demonstrated one of three major clusters. The first major cluster focussed on the article ‘Compassion Fatigue in Nurses: A Meta-synthesis’ (Paper 7 published in 2017) and resulted in 160 studies. Citings increased from 24 to 160 from 2019 to 2023, respectively. The primary disciplines referencing the article were nursing, business management and allied health professionals. The second major cluster focussed on the article ‘Birth Stories from South Africa: Voices Unheard’ (Paper 6), which was published in 2018. The study was cited two times in 2019 and 22 times in 2023. The third major cluster focussed on Ubuntu and included two articles: ‘An Integrative Review of Albertina Sisulu and Ubuntu – Relevance to Caring and Nursing’ (Paper 3 published in 2016) and ‘Ubuntu the Essence of Caring and Being – a Concept Analysis’ (Paper 14 published in 2019) with 41 total citing studies. Paper 16 (published in 2022) had no citations. All but two of the primary publications included the Fulbright recipient. Figure 2 demonstrates how studies are clustered.

### Study methods and design

The methodologic approach of the primary studies (*n* = 11) reviewed in the 2019 analyses (Downing et al. 2021) included three qualitative studies, two quantitative and six other methodologic studies (i.e. concept analyses, integrative review, etc.). Determination of the methodology used in primary works conducted after 2019 (*n* = 5) found the addition of one qualitative and quantitative approach each, as well as three other approaches (i.e. concept analyses, discourse).

The number of citing articles substantially increased from 42 (2013–2019) to 273 (2013–2023) with most citing studies having utilised a quantitative or qualitative methodology and most published in the *Journal of Clinical Nursing* and *Pain Management* journals. The increase in publications evinced the need to further explore the output and the impact of a Fulbright on nursing and other professions. Citations emanating from the primary referenced citations were primarily quantitative methodology (38%), followed by qualitative methodology (28%). Approximately, 38% were ‘other’ types (e.g. reviews, critical evaluations, concept analyses, discussions and opinions).

### Disciplinary affiliation

Most primary studies (*n* = 1) had multinational (*n* = 13, 81%) authorship; some had multidisciplinary authorship (*n* = 4, 25%). Citing studies reflected diverse disciplinary affiliations. The highest number of citing studies for referenced works were authored by scholars whose disciplines included nursing (*n* = 123), midwifery (*n* = 30), psychological or social and health sciences (*n* = 25), medicine and other healthcare (*n* = 16) and education (*n* = 11). Studies citing primary works also included multinational (*n* = 57, 22%) and multidisciplinary (*n* = 83, 32%) authorship.

Nursing scholars cited primary works 123 times in 2023 compared to 27 times in 2019. Midwifery citing increased from 11 to 30. Other categories not represented in the 2019 analytic work included engineering, information management systems and technology, political science, policy, rural health and economics.

### Geographic distribution

Referenced works in 2019 (Downing et al. 2021) were authored by scholars from the RSA, USA and the United Kingdom (UK), including Canada. All continents cited primary publications (*n* = 11) except South America. Ten of the publications had multinational authorship; three had multidisciplinary authorship. In our current study, the primary works (*n* = 16) added the engagement of a scholar from Eswatini. All but three of the original works had authorship from two or more countries.

**FIGURE 3:**
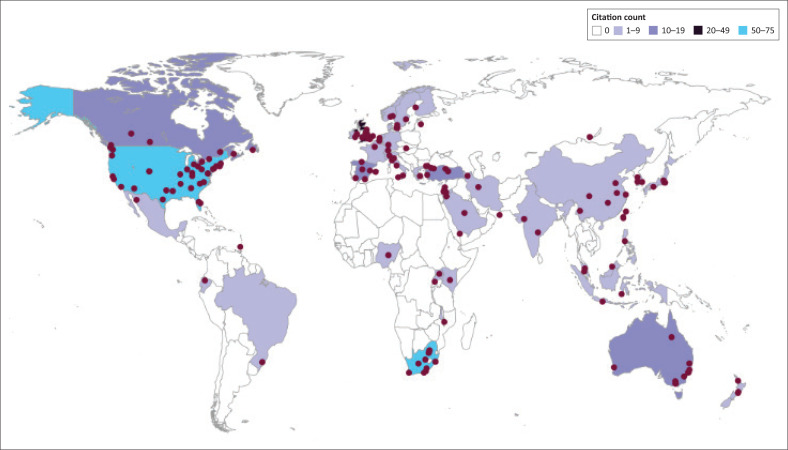
Global citation (*n* = 273) distribution citing 16 primary works (2013–2023).

**FIGURE 4:**
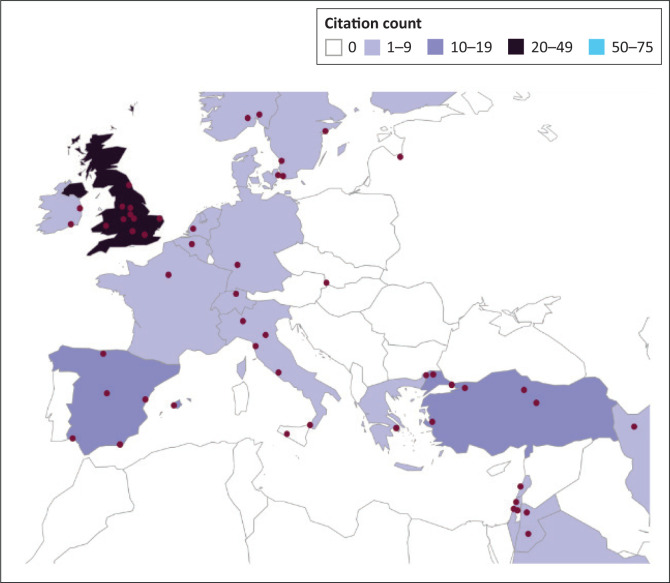
Citation (*n* = 273) distribution citing 16 primary works, detailed Europe and Middle East distribution.

The highest number of citing studies when considering all authors on secondary works were generated from the RSA (other than the rest of Africa) (*n* = 75), Africa (*n* = 7), USA (*n* = 65), Europe (*n* = 39), Asia (*n* = 36), Canada (*n* = 34), UK (England, Ireland only) (*n* = 32), Australia or New Zealand (*n* = 26), Middle East (*n* = 34), Central and South America (*n* = 6) and other countries (e.g. Caribbean) (*n* = 2). Figure 3 and Figure 4 demonstrate the geographic distribution for articles citing one of the 16 primary works published between 2013 and 2023. All countries increased their number of citations when compared to the 2019 analyses (Downing et al. 2021) with the UK and Canada increasing by 5.7- and 4-fold, respectively.

### Presentations

Only one of the original primary publications was a presentation (see Table 1, Paper 11). Secondary citations of the original studies found three presentations. However, only one presentation (see Table 2, Hlojeng & Makura 2020) had a published abstract that appeared in the Proceedings First database.

### Non-scientific downstream

Of the 16 primary publications, eight (50%) had no social media presence. There were alternative metrics for nine (56%) of the 16 primary sources. Specifically, usage metrics were available for nine of the publications, with abstract views for eight (50%) of the 16 primary sources (range 160–2592). There were link-outs for eight works, which ranged from 42 to 1717. Full-text views were noted for seven publications (range 0–1724), ‘export save’ was noted for eight (range 25–333), and ‘readers’ was noted for eight (range 14–143). There was one primary work picked up by five news outlets, and nine mentions of a work. Overall, nine primary citations had usage and citations; eight had captures. There were no reports of citations being referenced in videos (e.g. YouTube) or on research blogs. Only four of the primary works (Downing & Hastings-Tolsma 2016; Hastings-Tolsma & Nolte 2014; Hastings-Tolsma, Nolte & Temane 2018; Nolte et al. 2017) had been tweeted (range 3–32). Seven of the 16 works had been posted on Facebook but only one had any likes, shares, or comments. Of the 16 primary studies, eight (50%) had an AAS score (range 1–20, mean 4.87). Notably, all but two of the 16 studies had a DOI, which is required for the generation of an AAS.

When comparing our first analysis (Downing et al. 2021) with the current work, there was a social media presence for seven of 11 primary sources with three of those publications having tweets (range 23–32). Prior bibliometric work found Facebook likes, shares or comments unchanged (i.e. only one). The current work found usage, captures and citations of the 16 works at 56% (*n* = 9); these alternative metrics were found in 81% (*n* = 9) of the works analysed in 2019 (Downing et al. 2021). Usage metrics were available for nine sources and abstract views ranged from 65 to 2592. ‘Link-outs’ ranged from 18 to 1717 with ‘export save’ for seven publications (range 24–333) and ‘reader’ captures ranging from two to 143; there were no mentions in the original analyses. There were full-text views for six of the 11 primary publications (0–1724). Table 3 details the non-scientific downstream alternative metrics for the current study of 16 publications.

## Discussion

### Published works citing primary publications and growth trajectory

The 16 primary referenced works were cited in 273 publications. In contrast, 42 citations emanated from 11 primary citations when the analysis was conducted in 2019 (Downing et al. 2021). The 550% increase in citations gave evidence of solid growth with the bulk of growth at or after 2017 but before 2020. The large increase in citations was particularly influenced by the referencing of three primary works (Hastings-Tolsma et al. 2018; Nolte & Downing 2019; Nolte et al. 2017) (see Table 1).

**FIGURE 5:**
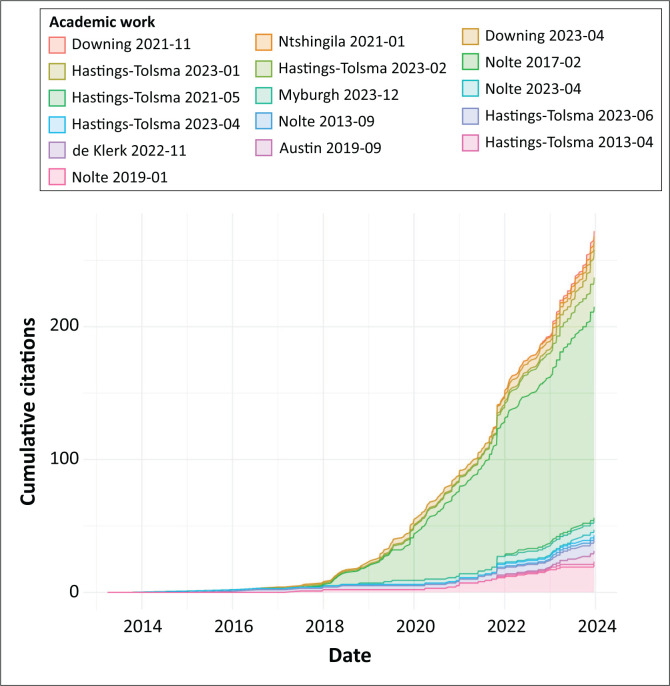
Persistence of reference studies (*N* = 16).

Citing of published studies tends to increase over time, reflecting a concept referred to as *persistence* (Nicoll et al. 2018). Long-term persistence was demonstrated in this research as noted in Figure 5. Cant and Cooper (2019) have noted that there is a ‘life cycle’ for publications with a steady accumulation of citations over the first 7 years, a constant high rate between years 4 and 7 and a reducing trend thereafter. Papers 2, 3, 7, 8, 9, 10 and 11 are 7 years or older. Seven of the 16 articles were available in open access (OA) journals. The highest citation of an article was 160 for the ‘Compassion Fatigue in Nurses’ A Meta-synthesis’ (Paper 6), which was available in an OA journal. The high citation count for that article demonstrated the development of science that requires a flourishing context (Achury-Saldana et al. 2022).

As noted, there was one primary work (Paper 16) that had no citing studies, which was likely the result of a recent publication in 2022. However, 11 other primary papers (1, 3, 4, 7, 8, 9, 10, 11, 12, 13, 15) had citation counts of eight or fewer. Seven of the 16 primary works were in OA journals (Papers 2, 6, 8, 10, 12, 13, 16) but only two of these primary works had high secondary citation counts (Papers 2 and 6). Publishing in OA journals has been demonstrated to increase citation counts though often in subsets (Langham-Putrow, Bakker & Riegelman 2021), as demonstrated in this research. Low secondary citations were undoubtedly influenced by those primary studies that had a narrow focus, lack of availability in OA journals and/or publication in low or unreported JIF journals. Such restrictions clearly impacted larger access and influence.

Finally, 12 of the 16 primary publications had self-citations; only four of the self-citations occurred within the first year following the publication of the primary work. There have been negative connotations with self-citing, but it has also been noted to be characteristic of highly productive authors (Mishra et al. 2018) with a self-citation rate of approximately 5% in the fields of science and technology (Kacem, Flatt & Mayr 2020). While our self-citation rate was 12% and higher than was anticipated, most of the citations resulted from student mentorship and were reflective of productive, sustained and leading-edge efforts (Cooke & Donaldson 2014).

### Impact of primary citations

To determine impact, cited primary works were examined for JIF, author-identified keywords, research methods, focus of major clusters, disciplinary affiliation, geographic origin of first authors and presentations (see Table 2). In addition, non-scientific downstream of primary citations was examined to determine influence on society (see Table 3).

#### Journal impact factor

The primary articles were referenced in journals with the highest JIF of 2.079 in the 2019 analysis (Downing et al. 2021). The highest JIF in the current study was 5.379 demonstrating a clear increase in being published in journals with a higher JIF and where there were a greater number of articles in the journal. Although JIF has long been regarded as the best and most impartial instrument for evaluating the prestige of a journal, there are still debates over how to calculate and interpret the score (Cooper 2015). There is agreement, however, that where there is an increase in the JIF, it indicates the journal is improving with greater numbers of researchers citing studies from the journal (Sharma et al. 2014). Journal Impact Factor, calculated annually, has many methodological flaws though academics are frequently evaluated against JIF for promotion, tenure and renumeration (Siler & Larivière 2022).

#### Keywords

Keyword frequency in citing references provided a good indication of the subject fields under study (Ellegaard & Wallin 2015). When comparing keywords between the initial study (Downing et al. 2021) and the current study, findings demonstrated that health, nursing and factors impacting nurses (e.g. compassion, fatigue, burnout, caring, Ubuntu and COVID-19) were significant objects of both bibliometric analyses. The keywords emanating from Paper 7 included compassion fatigue, burnout, vicarious trauma, traumatic stress, compassion satisfaction, compassionate care, coping and resilience. Twelve of the 160 studies identified COVID-19 as a keyword. Paper 6 had 22 citations and common keywords included labour, childbirth, humanisation, caring and uncaring encounters, birth companion, obstetric violence and neglect. Papers 3 and 16 focussed on the theme of Ubuntu and common keywords were caring and uncaring, Ubuntu, nursing and/or health professionals, community, quality of life and individualism. The frequency of these keywords reflected bursts of attention and underscored the strength of keywords and frequency of citation (Huang et al. 2020). As noted, the number of citations appearing in large numbers of studies gives proof of the influence of the original Fulbright award. Such notation documents the impact of the award in the expansion of foundational knowledge in a targeted area.

#### Focus of major clusters

The three clusters in this review of studies were ‘Compassion Fatigue in Nurses’: A Meta-synthesis’, ‘Birth Stories from South Africa: Voices Unheard’ and ‘Ubuntu’ (‘An Integrative Review of Albertina Sisulu and Ubuntu – Relevance to Caring and Nursing’ and ‘Ubuntu the Essence of Caring and Being – a Concept Analysis’). In toto, these three clusters reflected strong Fulbright and university alliance and were cited 273 times. The publications with the highest citation counts (Paper 6, 160 secondary citations; Paper 5, 22 secondary citations; Paper 2, 21 secondary citations; Paper 14, 21 secondary citations) had the greatest impact for a total of 224 secondary citations. These major clusters represent scientific topics and were easily recognised in the clusters of publications surrounding these works (Šubelj, Van Eck & Waltman 2016).

#### Research methodology

It was evident in the 16 primary works cited in 273 studies that Paper 6 (‘Compassion Fatigue in Nurses: A Meta-Synthesis’ by Nolte et al. 2017) had more citations. There was a plethora of methodologies that cited Paper 6 (i.e. qualitative, quantitative, discourse, systematic review, meta-analyses, cross-sectional and others); qualitative and quantitative methodologies were most common. There was demonstrated uptake in varied healthcare subspecialties with a significant impact on methodological design. Different research methods can use a variety of techniques and can also be used to find possible research partners for future studies. Ellegaard and Wallen (2015) argue that the use of different methods assists researchers to be aware of new trends in bibliometric methods.

#### Disciplinary affiliation

The comparison of disciplines with the number of citing studies for referenced works increased significantly. The diversification of disciplines in the current study was largely related to the expansion of disciplines researching COVID-19. Original research on coronavirus focussed on virology, medicine and infectious disease prior to the COVID-19 pandemic. After the pandemic, new research disciplines included sociology, psychology, environmental engineering, management and public administration (Abideen 2022). This mirrors the trend of increasing research on the social and psychological impacts of the pandemic as seen in the increase in compassion fatigue. Disciplines citing the Nolte et al. (2017) article on compassion fatigue increased from six in 2019 to 23 in 2023.

Nursing and midwifery remained the disciplines with the highest citing number of primary works (*n* = 180) and a significant increase from 27 citations in 2019. However, interdisciplinary collaborations increased to include industrial psychology and management, physiotherapy, occupational health and psychiatry. Of all citing studies, 32% (*n* = 83) were multidisciplinary. Publications demonstrated a pronounced expansion in the range of disciplines engaged in collaborative scientific efforts. Increased multidisciplinarity has been found to have a positive effect on impact and knowledge creation though there has been a suggestion that scientists may be more reluctant to site heterodox studies viewed as challenging or too groundbreaking (Yegros-Yegros, Rafols & D’Este 2015). Finally, 22% (*n* = 57) of citing studies had multinational authors. These transnational publications demonstrated a greater number of citations, which has also been noted in other bibliometric research (Adams & Gurney 2018). Multinational and multidisciplinary author affiliations are far-reaching and encourage cross-fertilisation of ideas, increasing impact and promoting development (Adams & Gurney 2018).

#### Geographic distribution

As in the original study (Downing et al. 2021), the RSA and USA were the two countries with the largest number of cited works. The RSA had the most citations in both analyses (*n* = 10, 2019; *n* = 64, 2023) with the USA again demonstrating the second largest number (*n* = 9, 2019; *n* = 44, 2023). This finding was likely because of the RSA and USA being the Fulbright host country and awardee country of origin, respectively.

Regardless, there was wide geographic distribution overall with an increase in the number of citations across all continents. Interestingly, distribution may have been influenced by the COVID-19 pandemic that began in the first quarter of 2020. The primary study by Nolte et al. (2017) discussed compassion fatigue and demonstrated the greatest persistence in the use of all primary works. The number of citations in the original analysis was 17 through 2019 (Downing et al. 2021); another 119 were added between 2019 and 2023 with approximately 20 articles directly addressing compassion fatigue and COVID-19. During the pandemic, the highest distribution of all research regarding compassion fatigue occurred in the USA, Canada, England and Australia (Yi et al. 2022).

The geographic growth in 2023 was likely influenced by seven OA studies, along with a number of tweets (*n* = 66), ResearchGate authors (*n* = 223) and authors’ primary country of affiliation of the RSA and USA. These factors likely had a positive influence on the number of citations (Chen et al. 2021; Gelzer et al. 2022; Tang et al. 2023). Finally, all primary articles were in English. This fact did not appear to negatively impact geographic growth apart from Central and South America where there were no citations of primary works in 2019 and three in the current analysis. These low citation counts were certainly influenced by language barriers, as well as the known exodus of scientists to the Global North (Basilio 2023).

#### Presentations

Only four of 19 presentations had a published abstract with a DOI, so it was not expected that there would be many references to these works. Presentations are intended for early dissemination and adoption of findings, among other goals such as networking and brainstorming (Oester et al. 2017). Researchers and conference organisers need to give greater attention to the widespread dissemination of conference abstracts, including to social media outlets with the assignment of a DOI number. The value of promissory abstracts is minimal, and it is suggested they be declined for conference presentation and dissemination (Novak & Puljak 2021). Finally, given the global explosion in conferences with an increase in ‘predatory conferences’ and the fact that there is no accreditation process for conferences, a bibliometric measure is needed to enable scholars to evaluate conference quality prior to submission of work (Makvandi, Nodehi & Tay 2021).

#### Non-scientific downstream

How a published work is promoted by publishers, authors and organisations through social media is important in advancing use (Gunaratne, Haghbayan & Coomes 2020). In this research, PlumX Analytics and Altmetrics were used to conduct alternative metrics for primary citations. While a wide variety of tools could have been used, the authors used these altmetric services for reasons of access and convenience. It should also be noted that the extent of use of social media (pick-up) does not reflect either the quality or impact of the work. However, the downstream use of publications does provide a complement to other conventional methods (e.g. JIF, h-, i10 or m-indices). Alternate metrics that track key usage metrics (e.g. views, mentions, shares and downloads) are known to be superior to more traditional filters in assessing scholarly impact (Patthi et al. 2017).

Of the 16 primary publications, eight (50%) had no social media presence, and it is concerning that there was so little social media or non-science visibility. Such promotion underscores lost opportunities for highlighting scholarly efforts and the potential for use by other scientists and interested parties. Where there is the use of social media in the promotion of research, improved citation is noted (Bardus et al. 2020; Klar et al. 2020).

There were alternate metrics of usage, captures and citations for nine (56%) of the 16 primary sources. Only four of the primary works (Downing & Hastings-Tolsma 2016; Hastings-Tolsma & Nolte 2014; Hastings-Tolsma et al. 2018; Nolte et al. 2017) had been tweeted (range 3–32). Seven of the 16 works had been posted on Facebook but only one had any likes, shares, and/or comments. Usage metrics were available for nine of the publications, with abstract views for eight (50%) of the 16 primary sources (range 160–2592). There were link-outs for eight works, which ranged from 42 to 1717. Full-text views were noted for seven publications (range 0–1724), ‘export save’ for eight (range 25–333) and ‘readers’ for eight (range 14–143). No mentions were found for any of the works in blog posts, newspapers, etc. Overall, nine primary citations had usage and citations; eight had captures.

Finally, only five of the 16 primary works had an AAS but other alternative metrics demonstrated that publications after 2016 and those that appeared in OA journals were most likely to be promoted through social media outlets. It is possible that other global events (e.g. pandemic and immigration patterns) were influential in pick-up and use by non-scientific communities. Results for downstream citations in non-scientific sources for the 16 primary sources are provided in Table 3.

Of note, the current study did not determine the extent to which individual authors who cited primary publications may have shared their work on social media, which is a limitation of this research. Where there are efforts to share information on social media, there is known to be a three-fold increase of shares by non-authors with greater than 50% downstream citations (Gunaratne et al. 2020). Similarly, where authors posted engagement on ResearchGate, 224 citations were produced, which far exceeded the number discerned from academic databases. Such citations demonstrate the value of social media.

In summary, it is important that both publishers and authors pay close attention to the value of disseminating research findings through social media outlets. Such outlets include social networking sites like X, Threads, YouTube video, research blogs, Google Scholar, ResearchGate, Academic.edu and LinkedIn, as well as multinational authorship with publication in OA journals (Gelzer et al. 2022; Tang et al. 2023). These sites have high numbers of active users that can be influential in the advancement of research findings. In fact, close to 70% of Americans get their news from social media because of ease of use (Shearer & Matsa 2018), and close to half of scientists follow social media to increase awareness of new discoveries and to further scientific discussions (Rainie, Funk & Anderson 2015). The value of using social media in the dissemination of publications is obvious. Such use can help scientists to have a stronger influence in a specific field (Gelzer et al. 2022) and transform how the authors communicate science to both colleagues and the broader audience (Iwasaki 2022).

### Limitations

There were several limitations in this research. Firstly, self-citations were included in this research. Including self-citations has become an increasingly worrisome practice as it may unduly distort bibliometric thresholds (Baccini, De Nicolao & Petrovich 2019). Secondly, citation counts do not assess quality; rather, they are essentially a measure of popularity and further analysis is indicated. Thirdly, more recent citations may have been missed as they were not yet cited in selected databases and works not in English may have provided substantive knowledge that was not assessed. Additionally, only works with a DOI were assessed in looking at non-scientific downstream influence; works without a DOI (e.g. books, magazine articles) may have had of significant impact without determination. Future research should consider these issues. Fourthly, the use of the first author as an indicator of the geographic origin of cited works fails to fully appreciate the reach of multi-authored teams from diverse institutions. The geographic distribution of works with multiple authors should be carefully considered in future work. Fifthly, the work conducted here would benefit from a robust examination of how primary sources were used in non-scientific outlets and how such usage impacted utilisation and influence. Sixthly, the altmetric services used to determine non-scientific downstream use of the primary publications were limited to PlumX Analytics and Altmetrics.com; other platforms should be explored such as ResearchGate, LinkedIn and Google Search.

### Future research

Consideration should be given to a robust examination of how the Fulbright award impacted other vital areas of engagement. This should include an examination of teaching and service involvement for both the Fulbright recipient and the host institution. It would also be useful to assess the impact of the award on generating future Fulbright applications that would further international partnerships. Replication of this research in the coming decade would be useful in providing further evidence of long-term impact. Our bibliometric work focussed on co-citation counts – or the number of times a primary source was cited. A useful strategy to consider going forward would be qualitative analysis examining the impact of publications (Cooper 2015). Qualitative assessment has been minimally used in bibliometric works but would be important to consider (Quaderi 2023). Finally, there continues to be a paucity of non-scientific downstream use of primary works – a recurring issue in the promotion of research in many fields. Evaluation of citation use in non-science journals and social media has not received significant attention and is an understudied but crucial area to consider.

### Implications

Findings from this research provide a compilation of the body of publications related to one research-teaching Fulbright award. The considerable growth in the use of primary publications – particularly over the past few years – underscores the impact and reach of a Fulbright award over the ensuing decade. Results provide evidence of the significance of such an award where scientists were committed to ongoing collaboration.

The primary implication of this study is that the outcome of a Fulbright award needs to demonstrate more than geographic relocation and connection of the awardee with other community members at the host site. Considerable resources are dedicated in support of a Fulbright scholar by the host institution, the awardee’s institution and the Fulbright Foundation. There needs to be clear expectations communicated to the awardee that the financial support provided has made a clear, sustained and demonstrable impact. Fulbright awardees have a responsibility to demonstrate the larger impact of the award. Bibliometrics is but one approach to documenting the impact and reach of cross-cultural engagement. Other approaches that could be considered include public recognition of work, evidence of change in practice and procurement of extramural grant funding.

## Conclusion

This study utilised co-citation bibliometric analyses to document the persistence and reach of 16 primary works that were published following a Fulbright award in 2012. The award created the opportunity for scientists from two universities in the USA and RSA to collaborate in scholarly efforts. From these collaborations between 2013 and 2023, 273 studies cited the usage of published works. While findings give solid evidence of a sustained connection between scientists, the research made no determination regarding the quality of the works. Assessment of both the quality of citing works, as well as persistence in use over the next decade, is needed to determine ongoing impact and reach. Additionally, while non-scientific downstream citations were sparse, they provided support regarding the impact and reach of the published works.

Persistence in maintaining collaborative relationships as an outgrowth of a Fulbright award is crucial to career success (Bu et al. 2018). While the quality of works emanating from this research was not assessed, persistent collaboration, multi-disciplinarity and team engagement gave evidence of the value of the Fulbright. Findings from this research paint a nuanced picture of how collaboration was of benefit to the two universities and can provide valuable insights to researchers, universities, policymakers and the Fulbright funding agency itself. Persistent scientific collaboration as an outgrowth of a Fulbright award is showcased in this project.
